# Supplementary material for: Caloric restriction delays age-related methylation drift
Source: Nat Commun. 2017 Sep 14;8:539. doi: 10.1038/s41467-017-00607-3 (PMC5599616; doi:10.1038/s41467-017-00607-3)
Supplement: Supplementary file 1 — Supplementary Information [file 41467_2017_607_MOESM1_ESM.pdf]

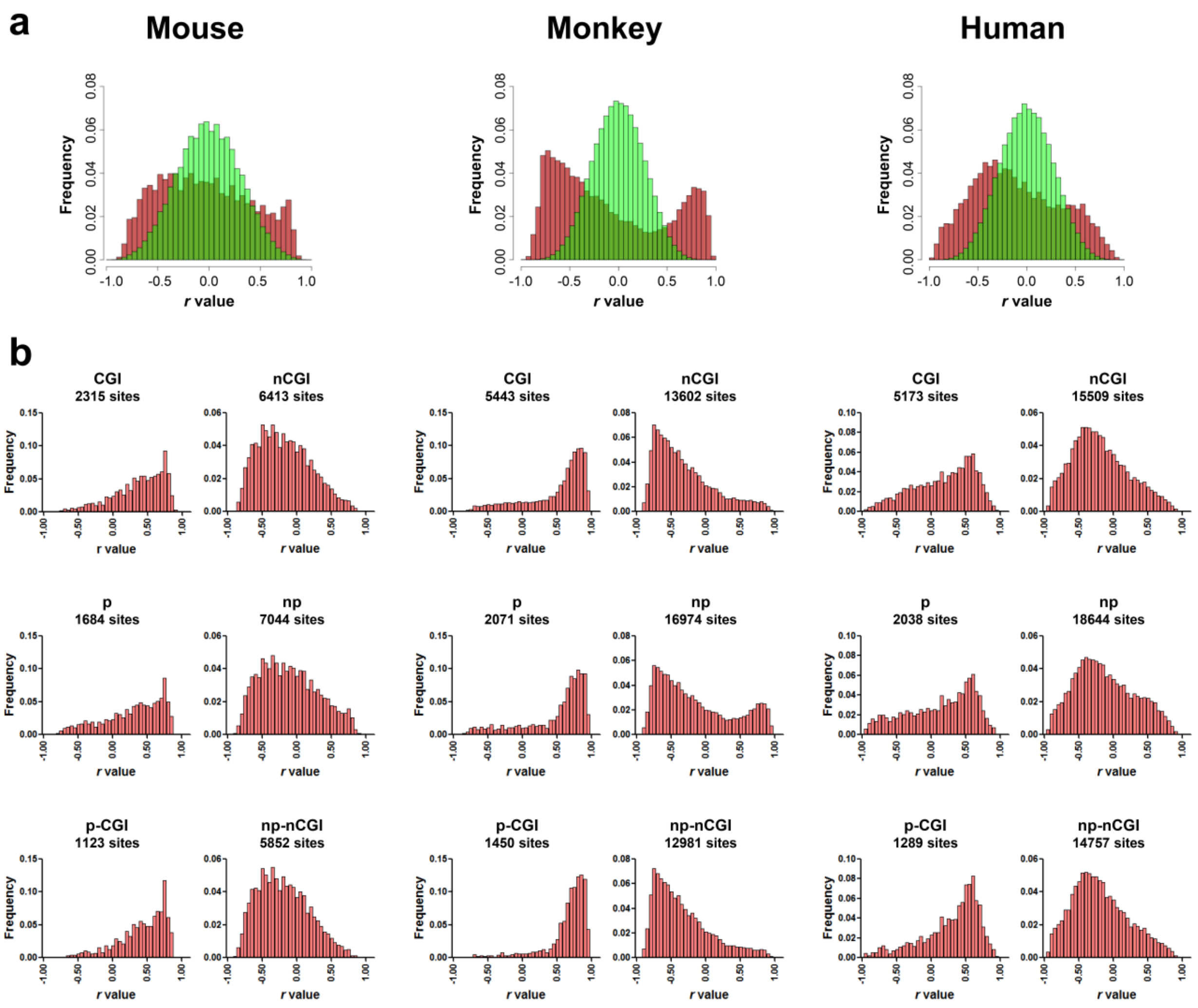

Supplementary Figure 1 | Distribution of *r*-values. (**a**) Histograms of observed (in red) and simulated (in green) *r*-values between age and methylation. Positive/negative value on x-axis means relative hypermethylation/hypomethylation in aged samples. The simulated *r*-values were derived from 1,000 random permutations for each species. (**b**) Histograms showing the distribution of *r*-values in different categories of CpG sites (CGI/non-CGI (nCGI), promoter (p)/non-promoter (np), and promoter-CGI/non-promoter-non-CGI).

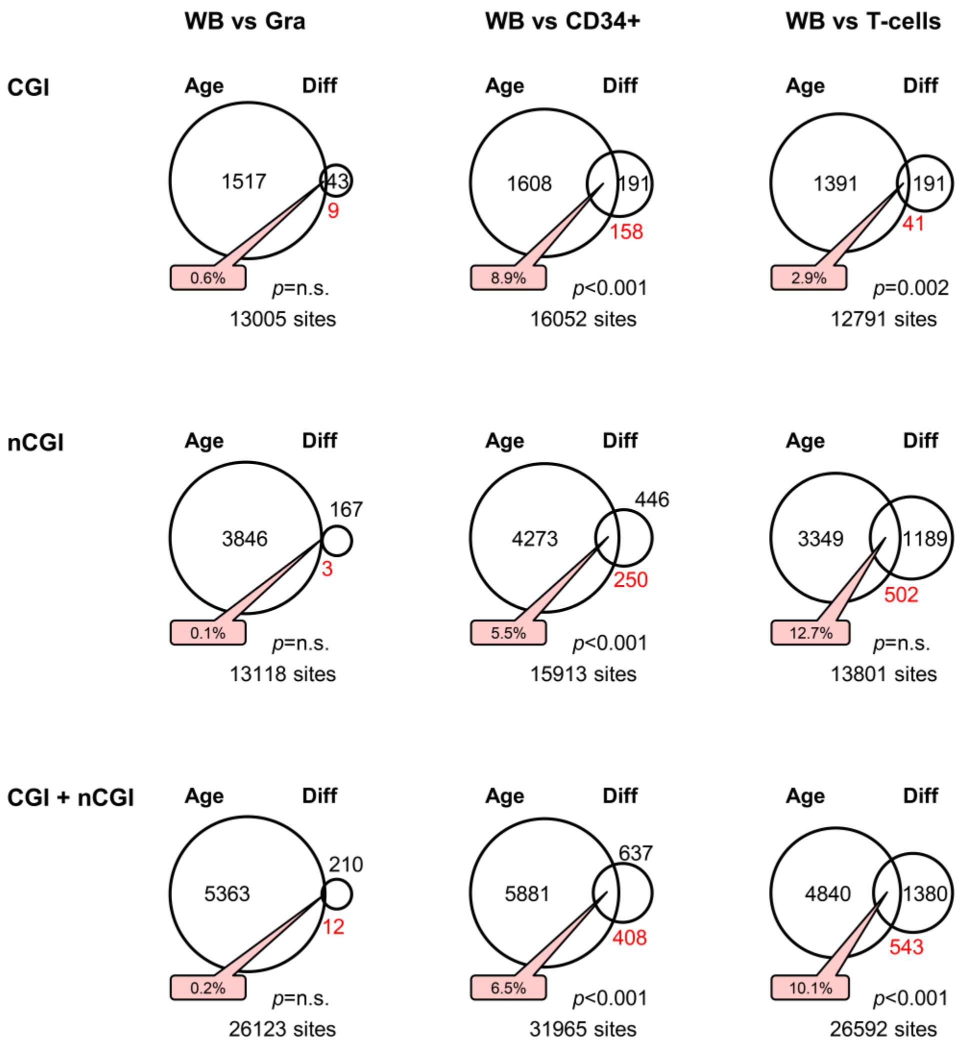

Supplementary Figure 2 | A limited effect of blood composition on age-related methylation. Area-proportional Venn diagrams of overlapped CpG sites between sites showing age-related methylation drift in whole blood and sites identified as differentially methylated in blood cell subtypes compared to whole blood. Red number represents the number of sites overlapping. Balloon shows the percentage of overlapped sites of age-related sites in whole blood. To identify the differentially methylated sites between whole blood and each blood cell type, we used sites with sequencing depth ≥ 100 reads in DREAM data among samples. Then, we compared the average of methylation % between the whole blood (n=16; age; 0-86y) and each blood cell type (granulocytes; n=6; age; unknown, CD34+ cells; n=2; age; unknown, T-cells; n=3; age; 19-21y) in each site and defined sites with methylation differences ≥2% (FDR<0.05) as differentially methylated sites. A Chi-square test using 2X2 tables (Supplementary Table 9) was used to calculate *p*-values for the significance of the overlaps. WB; Whole blood, Gra; Granulocytes, Age; Age-related sites in WB (both hyper and hypo-methylation drifts), Diff; Differentially methylated sites between whole blood and each cell sub-type.

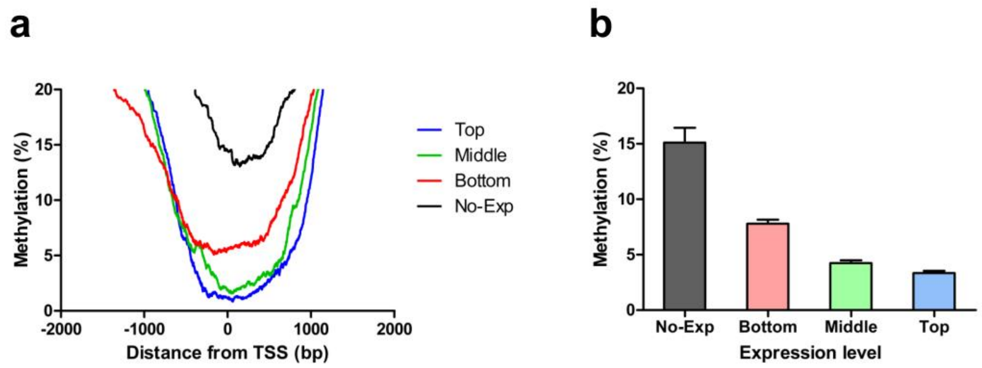

Supplementary Figure 3 | DNA methylation around TSS relative to gene expression. (**a**) Correlation of methylation profile with expression level in whole blood. Genes were divided into four groups based on gene expression levels detected by RNA-seq analyses (GSE53655). Each line represents the DNA methylation profile of different expression groups. The smoothed methylation levels for four representative groups are shown. Gene expression varies inversely with methylation level of TSS region. The most highly expressed genes (Top) exhibited the lowest methylation levels with the nadir of the hypomethylated “valley” centered ± 1kb from their TSS. As the gene expression decreased, the valley becomes shallower. (**b**) DNA methylation levels around the TSS (–1kb<TSS<+1kb) across four gene expression level bins. Bars represent standard errors of the mean. No-Exp; 536 sites (235 genes), Bottom; 3538 sites (1311 genes), Middle; 4839 sites (1904 genes), Top; 5070 sites (1882 genes).

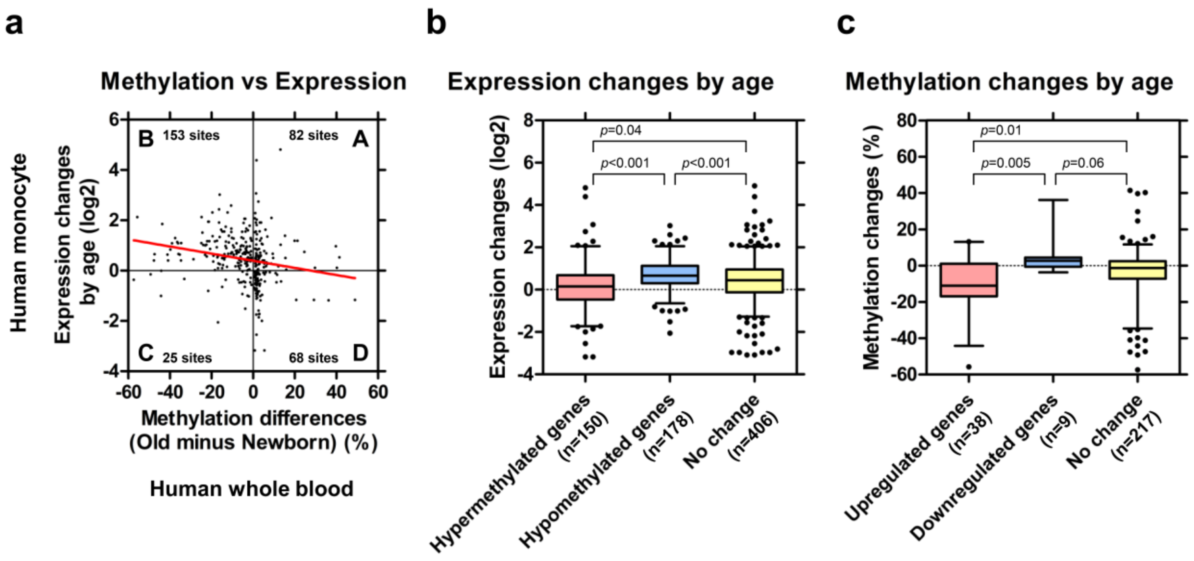

Supplementary Figure 4 | Inverse correlation between age-related methylation change and age-related expression change. (**a**) The quadrant plot of mRNA expression change versus methylation change in promoter regions. The x-axis represents methylation changes with age, and the y-axis represents log2 fold change of gene expression with age. The linear regression line is shown in red. The Spearman correlation coefficient r is −0.20 with p-value<0.001. The four quadrants shown are (A) hypermethylated and upregulated, (B) hypomethylated and upregulated, (C) hypomethylated and downregulated and (D) hypermethylated and downregulated. (**b**) Box plot of age-related expression changes (log2 ratio). The genes were subdivided into age-related hypermethylated, age-related hypomethylated and "no change" groups. The box-whisker plot shows the mean (horizontal line in box), 25–75 percentiles (box), 5–95 percentiles (whiskers), and values outside this range as black dots. p-values were obtained using the unpaired t-test with Welch's correction. (**c**) Box plot of age-related methylation changes (%). The genes were subdivided into age-related upregulated, age-related downregulated and "no change" groups. The box-whisker plot shows the mean (horizontal line in box), 25–75 percentiles (box), 5–95 percentiles (whiskers), and values outside this range as black dots. p-values were obtained using the unpaired t-test with Welch's correction.

Mouse

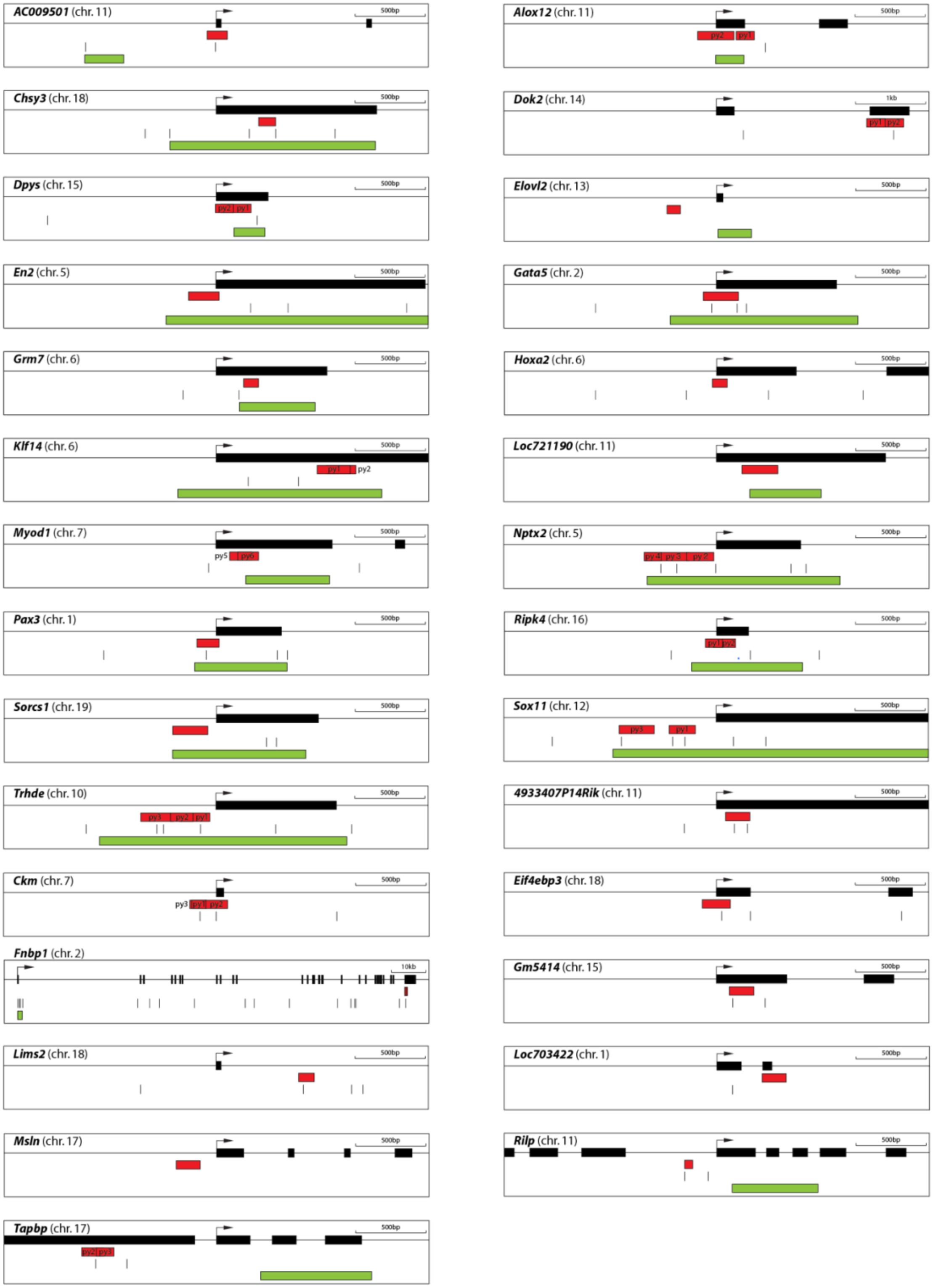
AC009501 (chr. 11)
Alox12 (chr. 11)
Chsy3 (chr. 18)
Dok2 (chr. 14)
Dpys (chr. 15)
Elovl2 (chr. 13)
En2 (chr. 5)
Gata5 (chr. 2)
Grm7 (chr. 6)
Hoxa2 (chr. 6)
Klf14 (chr. 6)
Loc721190 (chr. 11)
Myod1 (chr. 7)
Nptx2 (chr. 5)
Pax3 (chr. 1)
Ripk4 (chr. 16)
Sorcs1 (chr. 19)
Sox11 (chr. 12)
Trhde (chr. 10)
4933407P14Rik (chr. 11)
Ckm (chr. 7)
Eif4ebp3 (chr. 18)
Fnbp1 (chr. 2)
Gm5414 (chr. 15)
Lims2 (chr. 18)
Loc703422 (chr. 1)
Msln (chr. 17)
Rilp (chr. 11)
Tapbp (chr. 17)
500bp
1kb
10kb

Monkey

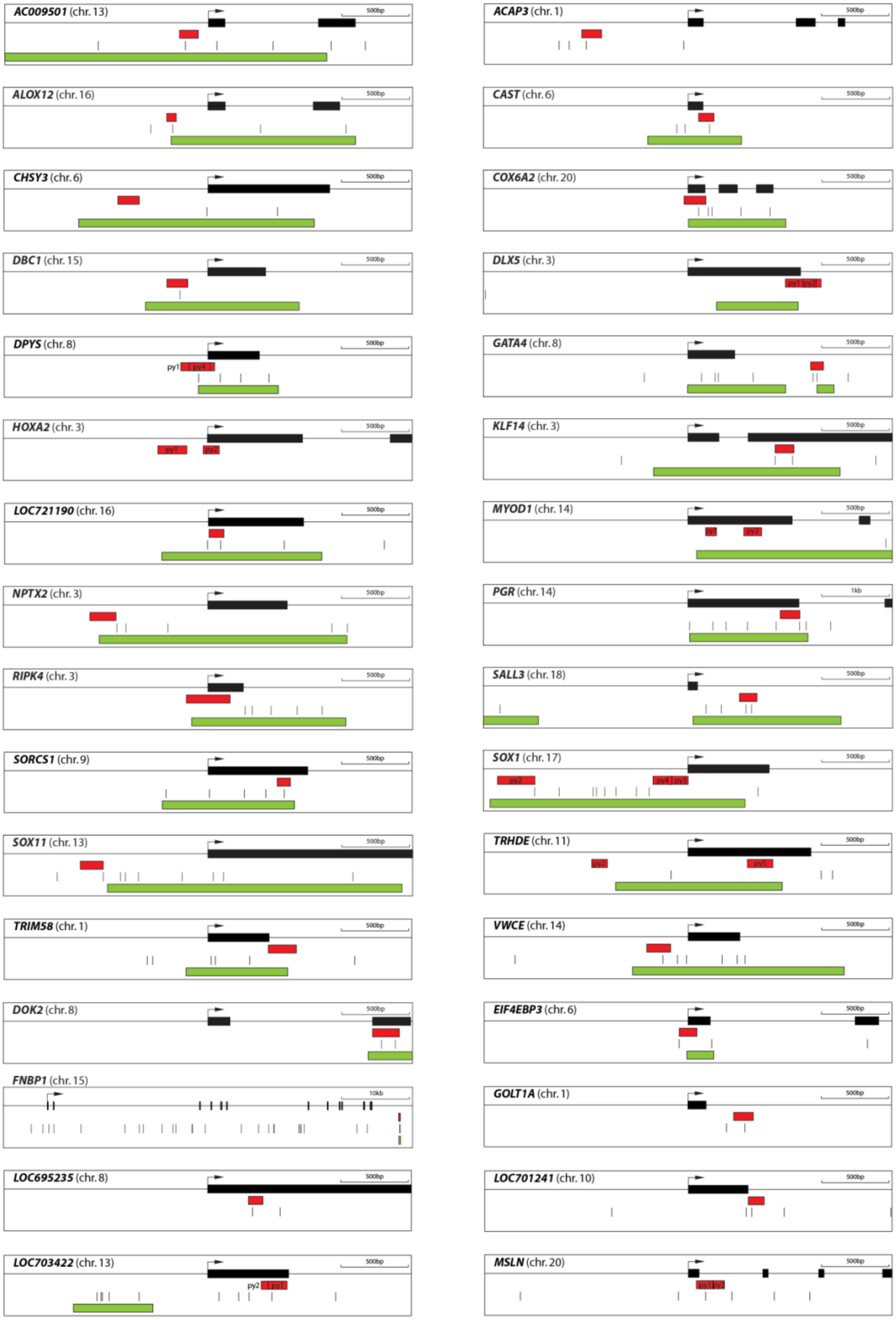
AC009501 (chr. 13)
ACAP3 (chr. 1)
ALOX12 (chr. 16)
CAST (chr. 6)
CHSY3 (chr. 6)
COX6A2 (chr. 20)
DBC1 (chr. 15)
DLX5 (chr. 3)
DPYS (chr. 8)
GATA4 (chr. 8)
HOXA2 (chr. 3)
KLF14 (chr. 3)
LOC721190 (chr. 16)
MYOD1 (chr. 14)
NPTX2 (chr. 3)
PGR (chr. 14)
RIPK4 (chr. 3)
SALL3 (chr. 18)
SORCS1 (chr. 9)
SOX1 (chr. 17)
SOX11 (chr. 13)
TRHDE (chr. 11)
TRIM58 (chr. 1)
VWCE (chr. 14)
DOK2 (chr. 8)
EIF4EBP3 (chr. 6)
FNBP1 (chr. 15)
GOLT1A (chr. 1)
LOC695235 (chr. 8)
LOC701241 (chr. 10)
LOC703422 (chr. 13)
MSLN (chr. 20)
500bp
1kb
10kb

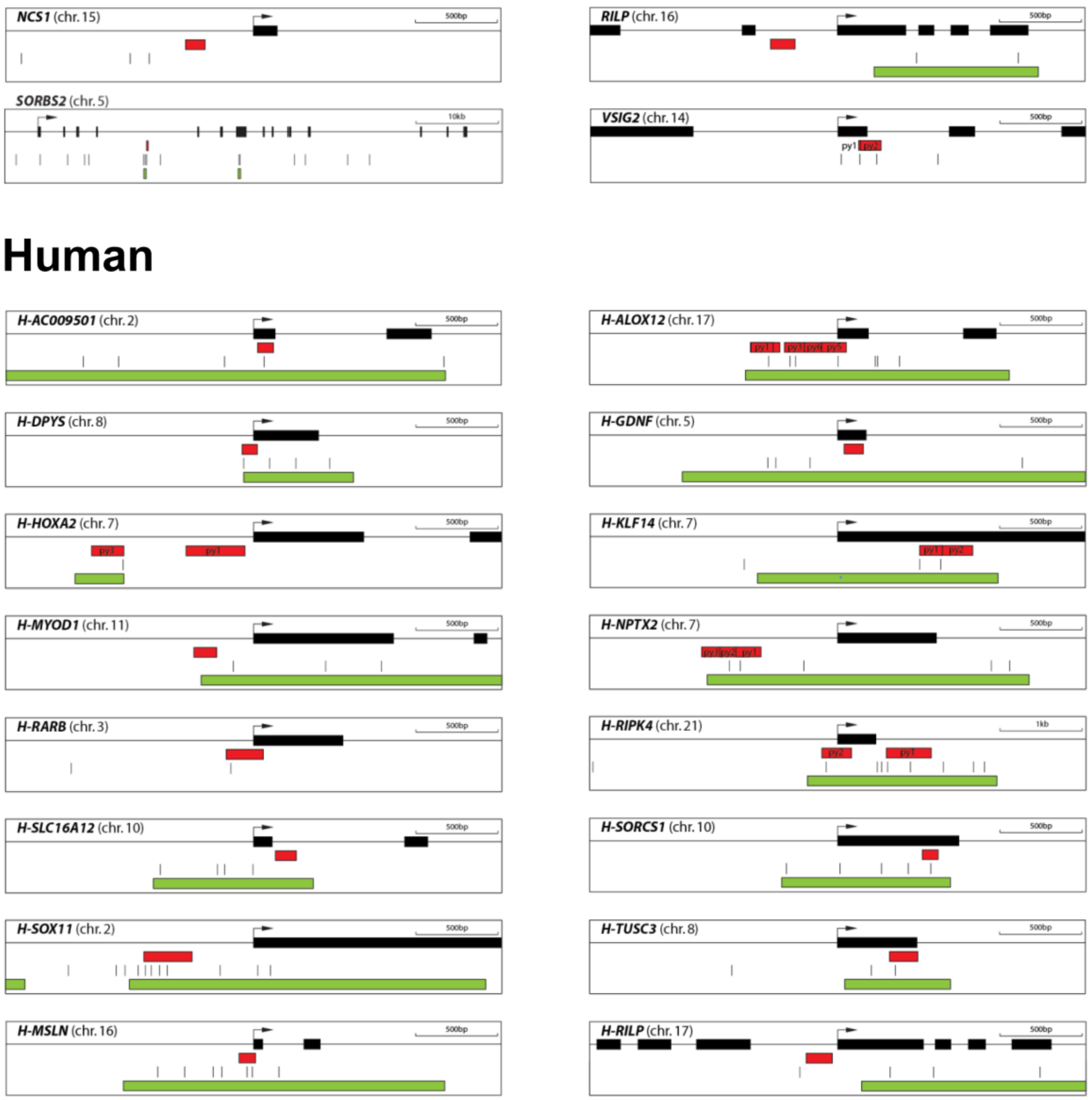

Supplementary Figure 5 | Gene structure and CpG sites analyzed in mouse, monkey and human by bisulfite pyrosequencing. Maps represent 3 kb of sequence around CpG islands (green boxes) and exons (black boxes) of genes analyzed in this study. Short vertical bars represent CCCGGG sites targeted by DREAM. Arrows point to transcriptional start sites. Red boxes represent regions amplified for bisulfite pyrosequencing.

a Mouse

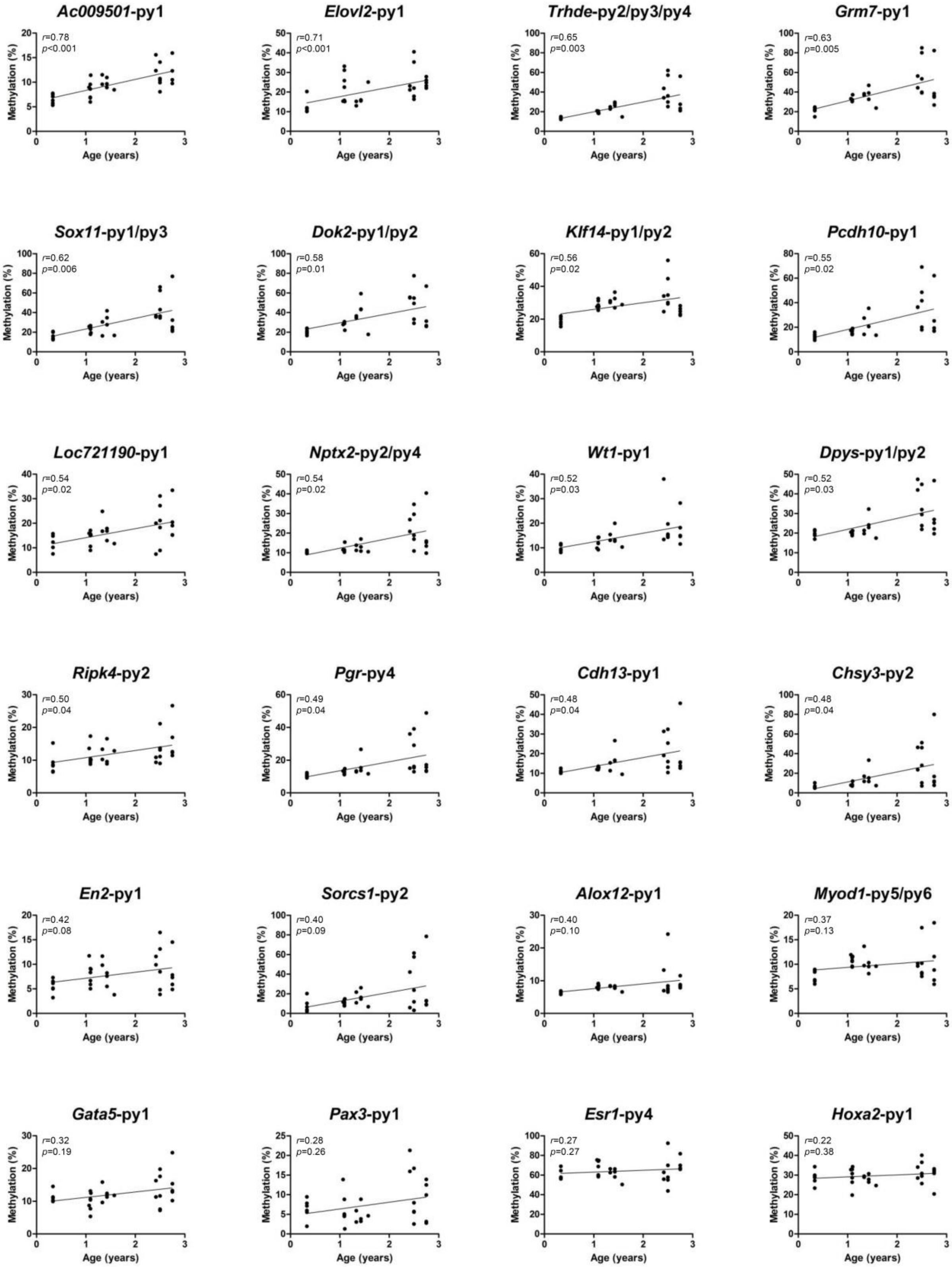
Ac009501-py1
r=0.78
p<0.001
Elovl2-py1
r=0.71
p<0.001
Trhde-py2/py3/py4
r=0.65
p=0.003
Grm7-py1
r=0.63
p=0.005
Sox11-py1/py3
r=0.62
p=0.006
Dok2-py1/py2
r=0.58
p=0.01
Klf14-py1/py2
r=0.56
p=0.02
Pcdh10-py1
r=0.55
p=0.02
Loc721190-py1
r=0.54
p=0.02
Nptx2-py2/py4
r=0.54
p=0.02
Wt1-py1
r=0.52
p=0.03
Dpys-py1/py2
r=0.52
p=0.03
Ripk4-py2
r=0.50
p=0.04
Pgr-py4
r=0.49
p=0.04
Cdh13-py1
r=0.48
p=0.04
Chsy3-py2
r=0.48
p=0.04
En2-py1
r=0.42
p=0.08
Sorcs1-py2
r=0.40
p=0.09
Alox12-py1
r=0.40
p=0.10
Myod1-py5/py6
r=0.37
p=0.13
Gata5-py1
r=0.32
p=0.19
Pax3-py1
r=0.28
p=0.26
Esr1-py4
r=0.27
p=0.27
Hoxa2-py1
r=0.22
p=0.38
Methylation (%)
Age (years)

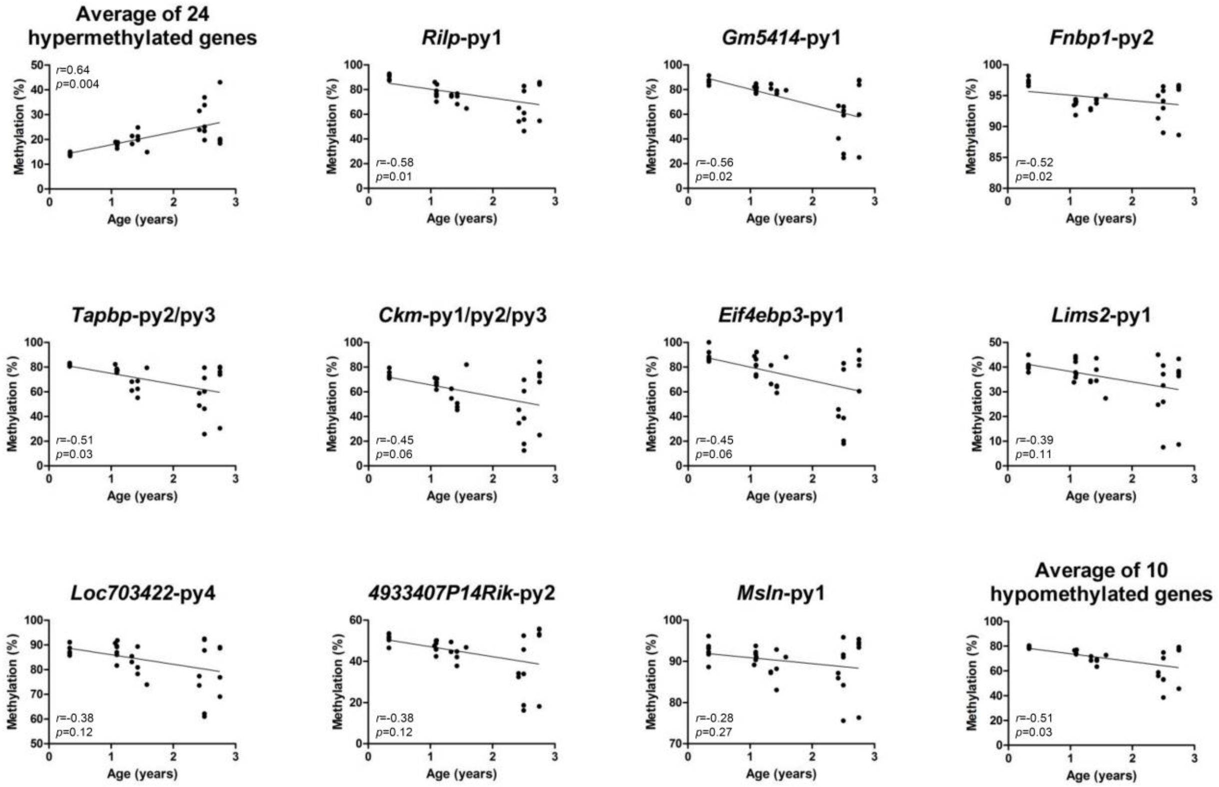
Average of 24 hypermethylated genes
r=0.64
p=0.004
Rilp-py1
r=-0.58
p=0.01
Gm5414-py1
r=-0.56
p=0.02
Fnbp1-py2
r=-0.52
p=0.02
Tapbp-py2/py3
r=-0.51
p=0.03
Ckm-py1/py2/py3
r=-0.45
p=0.06
Eif4ebp3-py1
r=-0.45
p=0.06
Lims2-py1
r=-0.39
p=0.11
Loc703422-py4
r=-0.38
p=0.12
4933407P14Rik-py2
r=-0.38
p=0.12
Msln-py1
r=-0.28
p=0.27
Average of 10 hypomethylated genes
r=-0.51
p=0.03
Methylation (%)
Age (years)

b Monkey

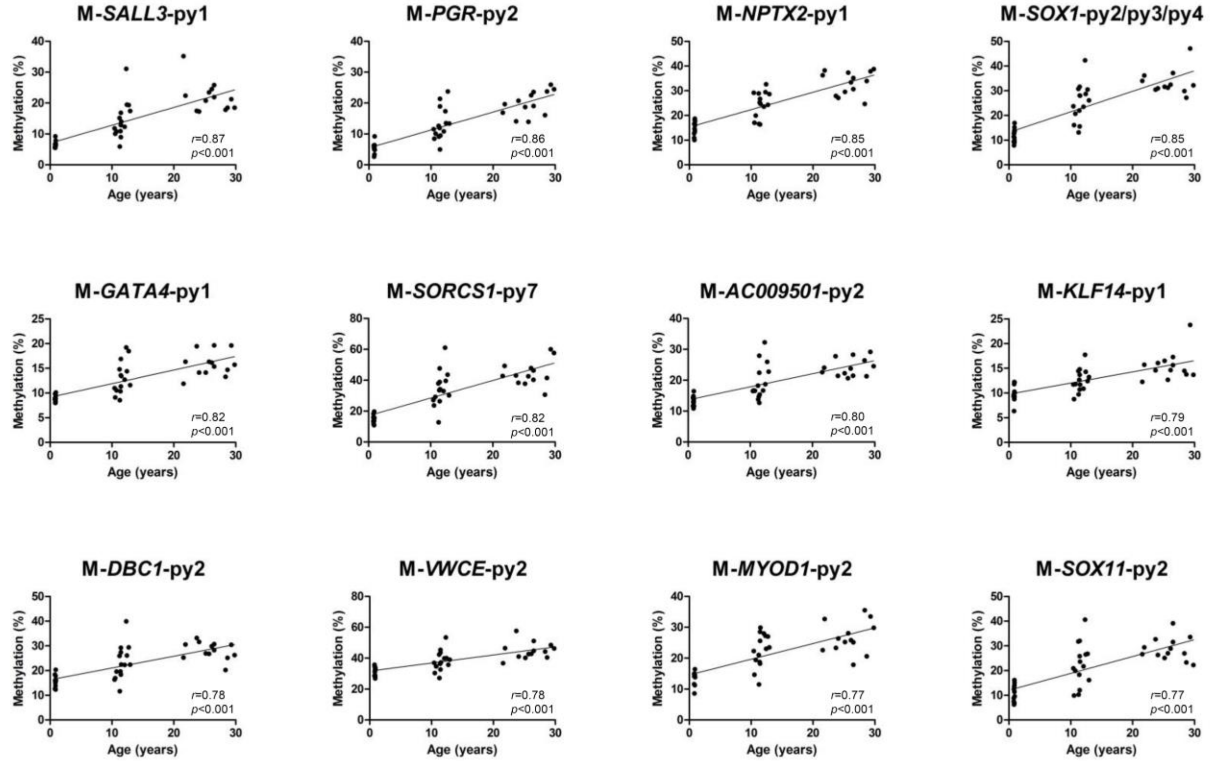
M-SALL3-py1
r=0.87
p<0.001
M-PGR-py2
r=0.86
p<0.001
M-NPTX2-py1
r=0.85
p<0.001
M-SOX1-py2/py3/py4
r=0.85
p<0.001
M-GATA4-py1
r=0.82
p<0.001
M-SORCS1-py7
r=0.82
p<0.001
M-AC009501-py2
r=0.80
p<0.001
M-KLF14-py1
r=0.79
p<0.001
M-DBC1-py2
r=0.78
p<0.001
M-VWCE-py2
r=0.78
p<0.001
M-MYOD1-py2
r=0.77
p<0.001
M-SOX11-py2
r=0.77
p<0.001
Methylation (%)
Age (years)

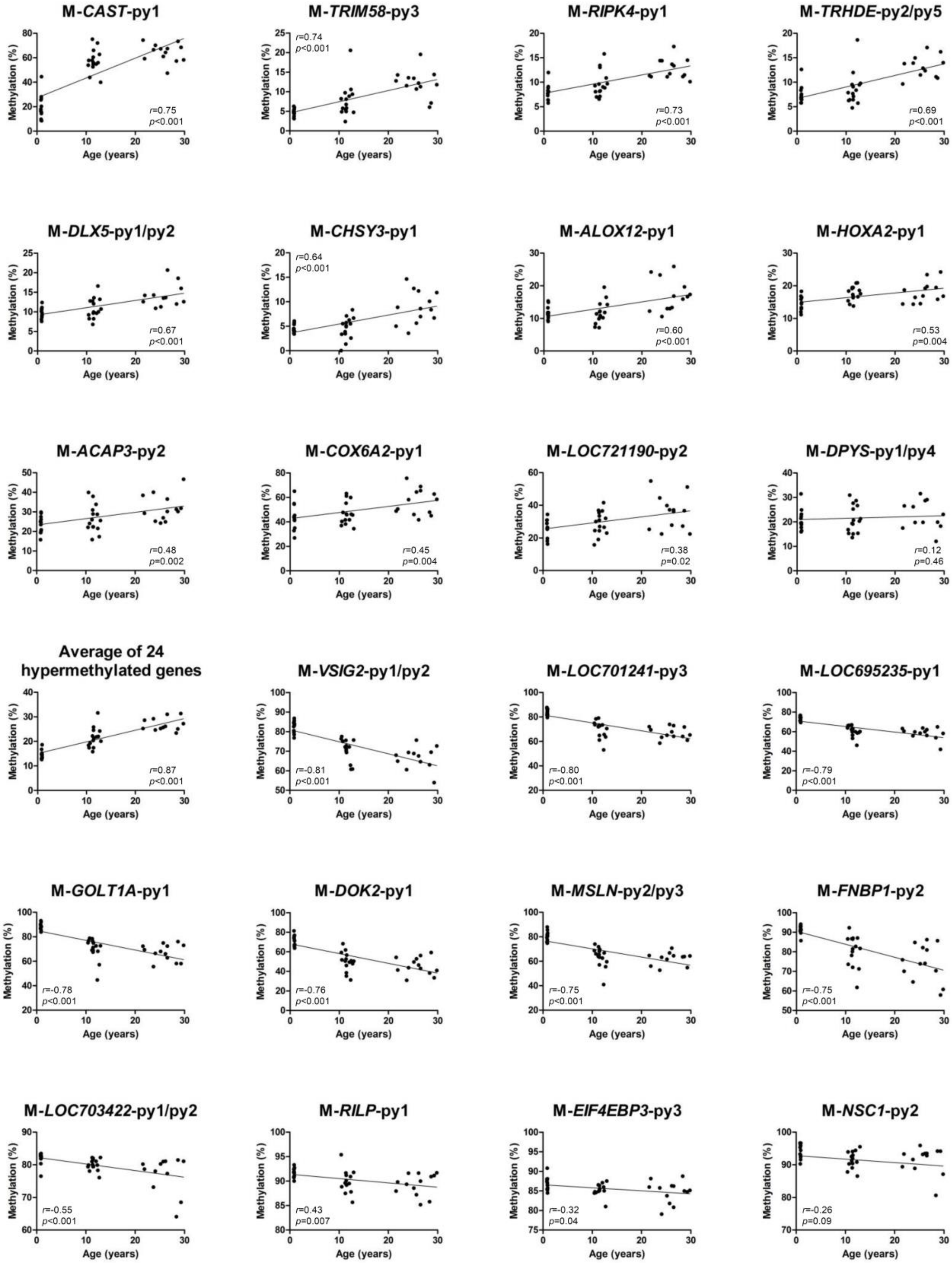
M-CAST-py1
r=0.75
p<0.001
M-TRIM58-py3
r=0.74
p<0.001
M-RIPK4-py1
r=0.73
p<0.001
M-TRHDE-py2/py5
r=0.69
p<0.001
M-DLX5-py1/py2
r=0.67
p<0.001
M-CHSY3-py1
r=0.64
p<0.001
M-ALOX12-py1
r=0.60
p<0.001
M-HOXA2-py1
r=0.53
p=0.004
M-ACAP3-py2
r=0.48
p=0.002
M-COX6A2-py1
r=0.45
p=0.004
M-LOC721190-py2
r=0.38
p=0.02
M-DPYS-py1/py4
r=0.12
p=0.46
Average of 24 hypermethylated genes
r=0.87
p<0.001
M-VSIG2-py1/py2
r=-0.81
p<0.001
M-LOC701241-py3
r=-0.80
p<0.001
M-LOC695235-py1
r=-0.79
p<0.001
M-GOLT1A-py1
r=-0.78
p<0.001
M-DOK2-py1
r=-0.76
p<0.001
M-MSLN-py2/py3
r=-0.75
p<0.001
M-FNBP1-py2
r=-0.75
p<0.001
M-LOC703422-py1/py2
r=-0.55
p<0.001
M-RILP-py1
r=0.43
p=0.007
M-EIF4EBP3-py3
r=-0.32
p=0.04
M-NSC1-py2
r=-0.26
p=0.09
Methylation (%)
Age (years)

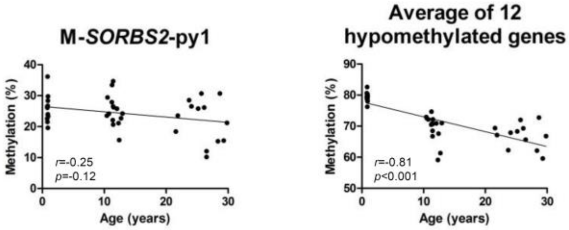
M-SORBS2-py1
r=-0.25
p=-0.12
Average of 12 hypomethylated genes
r=-0.81
p<0.001
Methylation (%)
Age (years)

c Human

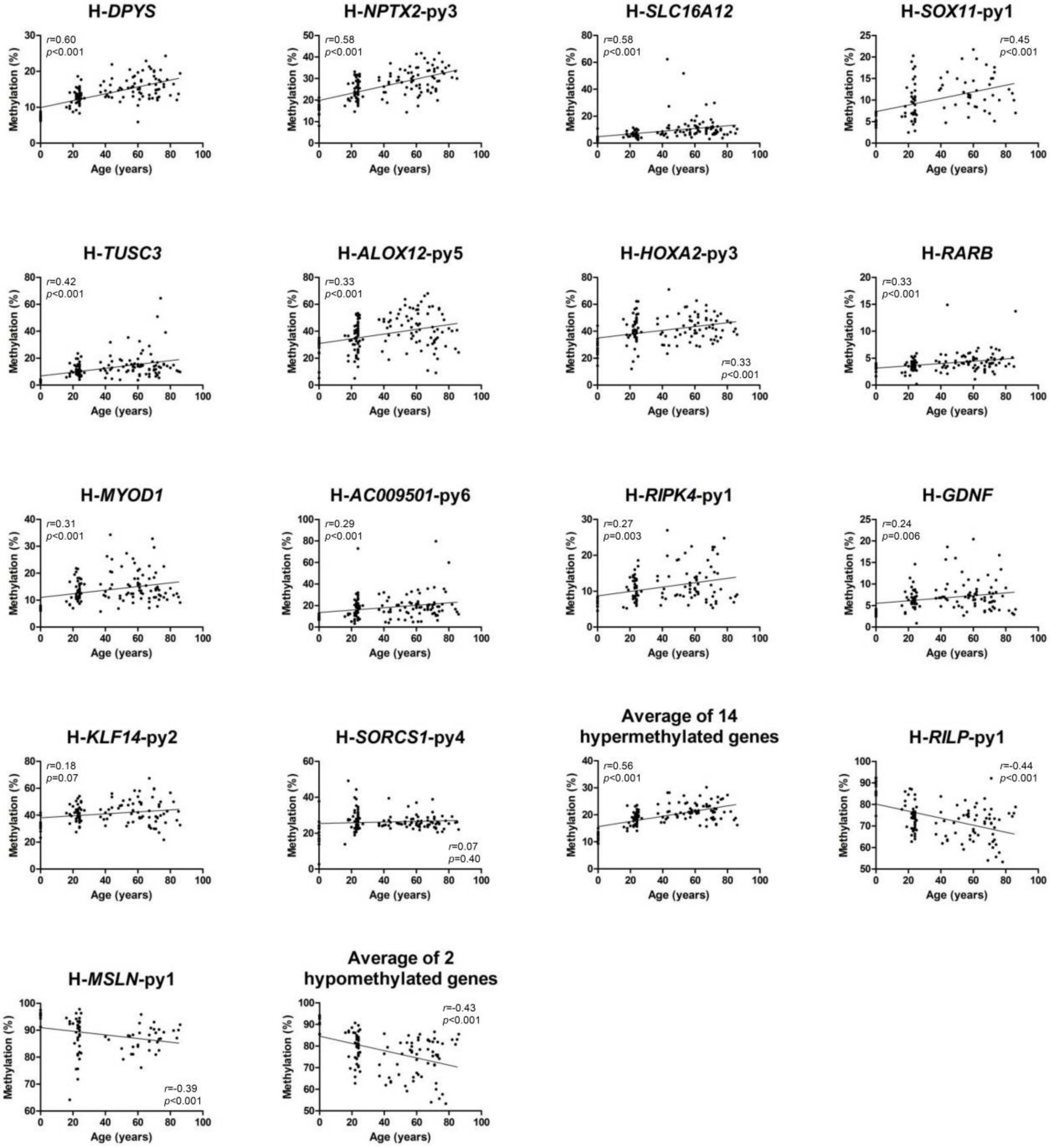
H-DPYS
r=0.60
p<0.001
H-NPTX2-py3
r=0.58
p<0.001
H-SLC16A12
r=0.58
p<0.001
H-SOX11-py1
r=0.45
p<0.001
H-TUSC3
r=0.42
p<0.001
H-ALOX12-py5
r=0.33
p<0.001
H-HOXA2-py3
r=0.33
p<0.001
H-RARB
r=0.33
p<0.001
H-MYOD1
r=0.31
p<0.001
H-AC009501-py6
r=0.29
p<0.001
H-RIPK4-py1
r=0.27
p=0.003
H-GDNF
r=0.24
p=0.006
H-KLF14-py2
r=0.18
p=0.07
H-SORCS1-py4
r=0.07
p=0.40
Average of 14 hypermethylated genes
r=0.56
p<0.001
H-RILP-py1
r=-0.44
p<0.001
H-MSLN-py1
r=-0.39
p<0.001
Average of 2 hypomethylated genes
r=-0.43
p<0.001
Methylation (%)
Age (years)

Supplementary Figure 6 | Age-related genes in mouse, monkey and human. Association of the percentages of methylated cytosines in the samples as obtained from bisulfite pyrosequencing (y-axis) with age (x-axis) (**a**; mice, **b**; monkeys, and **c**; humans). Each dot corresponds to one individual (mouse; young, n=6, middle, n=13, old, n=12, monkey; infant, n=12, middle, n=15, old, n=12, human; newborn, n=13, young, n=54, middle, n=27, old, n=45). Spearman *r* values and *p*-values (two-tailed) were listed in Supplementary Tables 14–16.

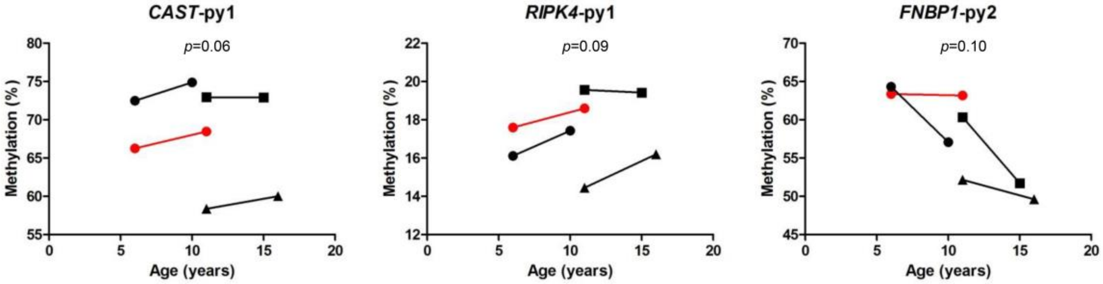

Supplementary Figure 7 | Intra-individual changes in methylation over time. The methylation levels of four genes were explored by bisulfite pyrosequencing analyses. The symbols connected by lines represent samples derived from one individual in different time points. Black and red symbols/lines represent AL and CR animal(s), respectively. Each point represents the mean of triplicate experiments (PCR reactions). Paired t-test was used for *p*-value calculation.

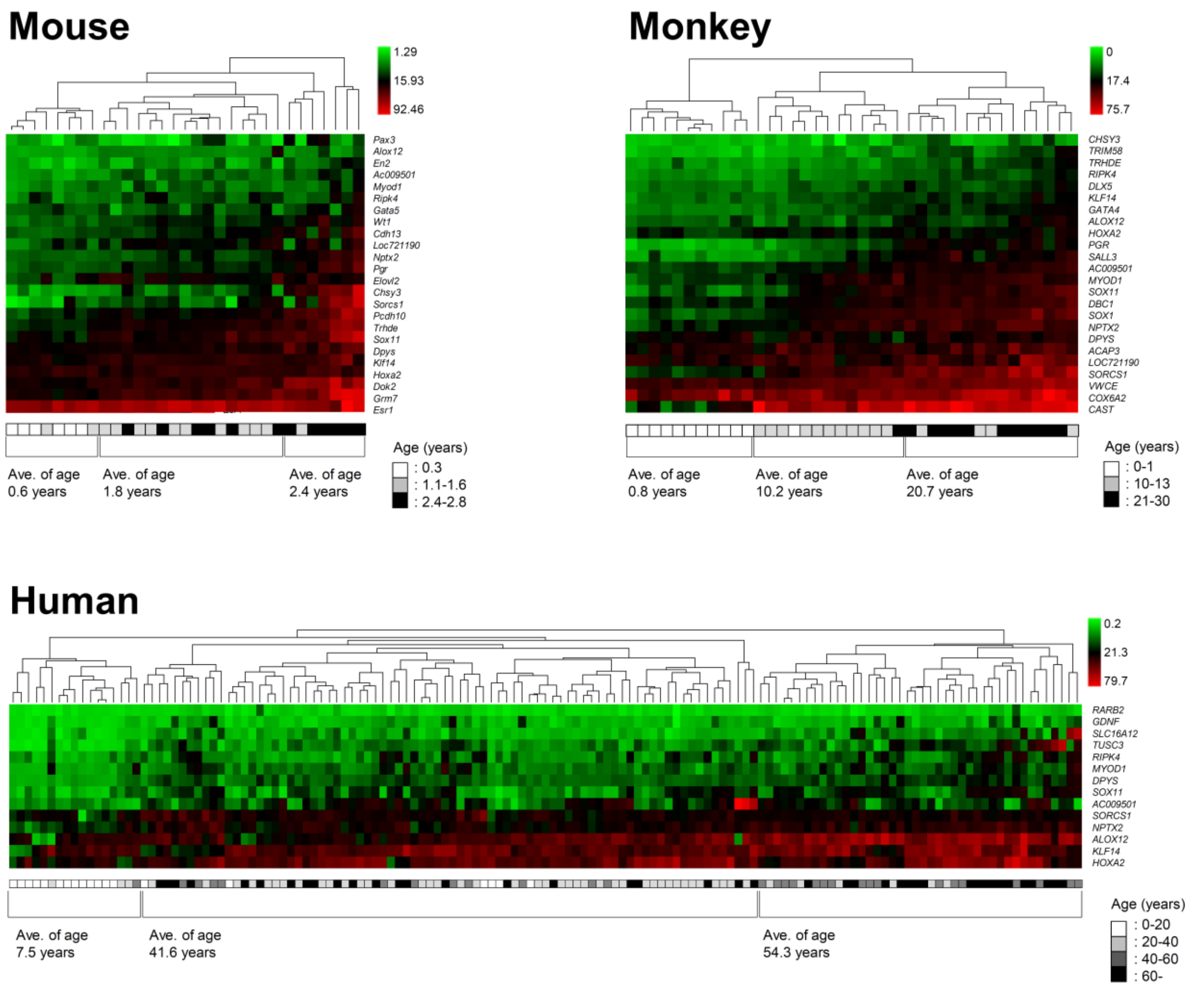

Supplementary Figure 8 | Unsupervised clustering analysis of bisulfite pyrosequencing results. Samples are shown with labeling of the corresponding ages. Samples were clustered by unsupervised hierarchical clustering of bisulfite pyrosequencing methylation values of genes. The green to red scale indicates the methylation percentage.

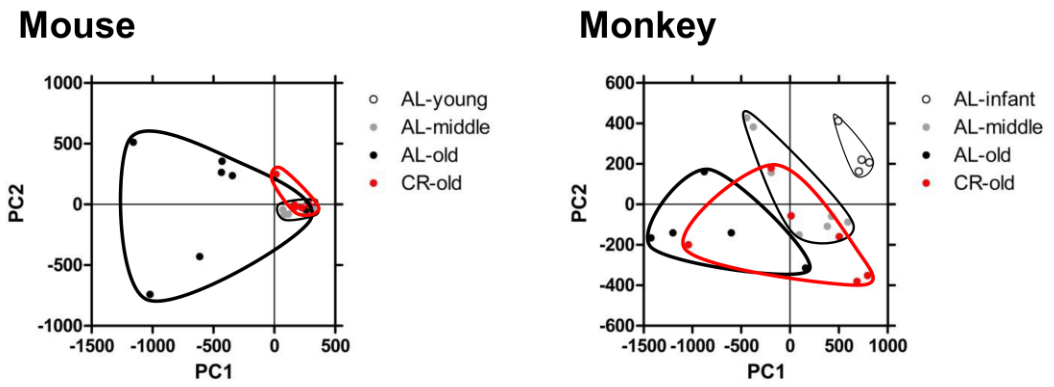

Supplementary Figure 9 | Principal component analysis of DNA methylation profiles detected by DREAM. Principal component analysis was performed on the DNA methylation data for CpG sites in all genomic regions (CGI + nCGI) (sequencing depth ≥100 reads in 75% of samples). Samples are plotted using the first two principal components (PC). The color codes of samples are shown on the right. Number of individuals (n) was as follows: mouse; AL-young, n=5, AL-middle, n=7, AL-old, n=7, CR-old, n=5, monkey; AL-infant, n=4, AL-middle, n=7, AL-old, n=5, CR-old, n=6.

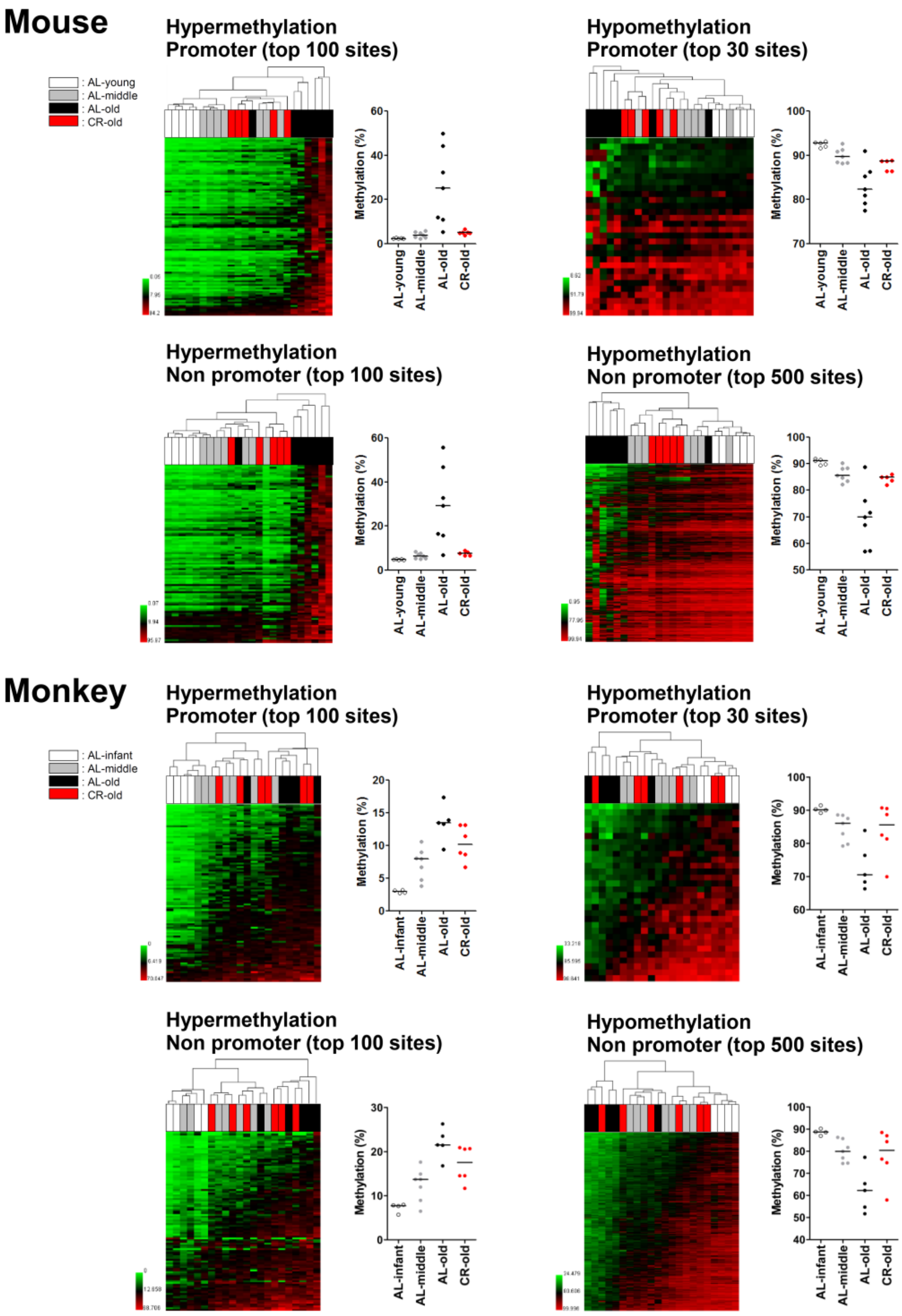

Supplementary Figure 10 | Clustering analyses of age-related hypermethylated and age-related hypomethylated CpG sites in mice and monkeys. Only CpG sites that showed age-related methylation were clustered. A total of top 100, 30, 100 and 500 sites were used that showed age-related hyper- or hypo-methylation in old animals compared with that of young age animals in average DNA methylation values by DREAM assay in promoter regions and non-promoter regions, respectively. The green to red scales indicate the methylation percentage. Shown in figures next to clustering are plots for the average methylation values in each animal group. The bar in the graphs represents the median.

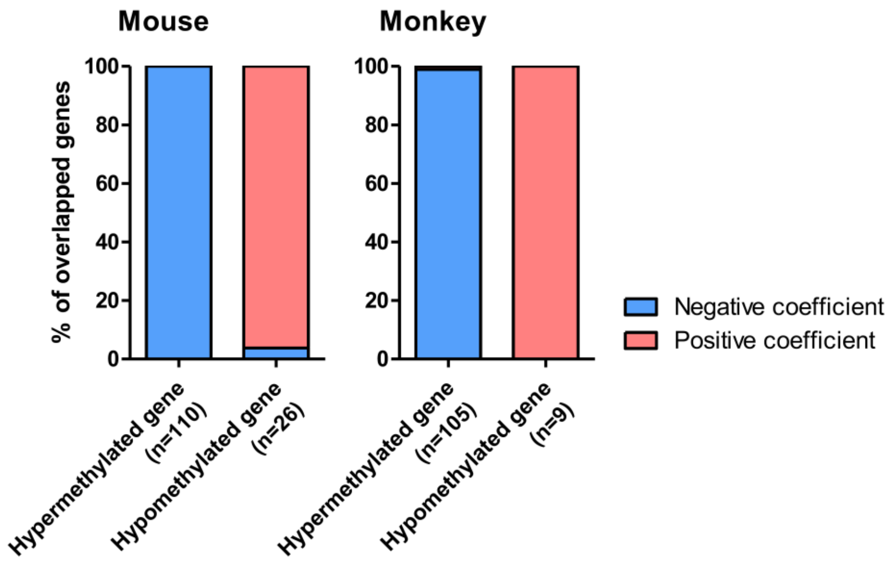

Supplementary Figure 11 | Overlap between genes affected by CR and genes showing age-related methylation. Coefficient negative means that CR tends to decrease aging methylation drift and vice versa. To test if CR tends to decrease or increase aging methylation drift, binomial test with probability 0.5 was performed for each bar. The *p*-values are smaller than 0.01 for all the bars.

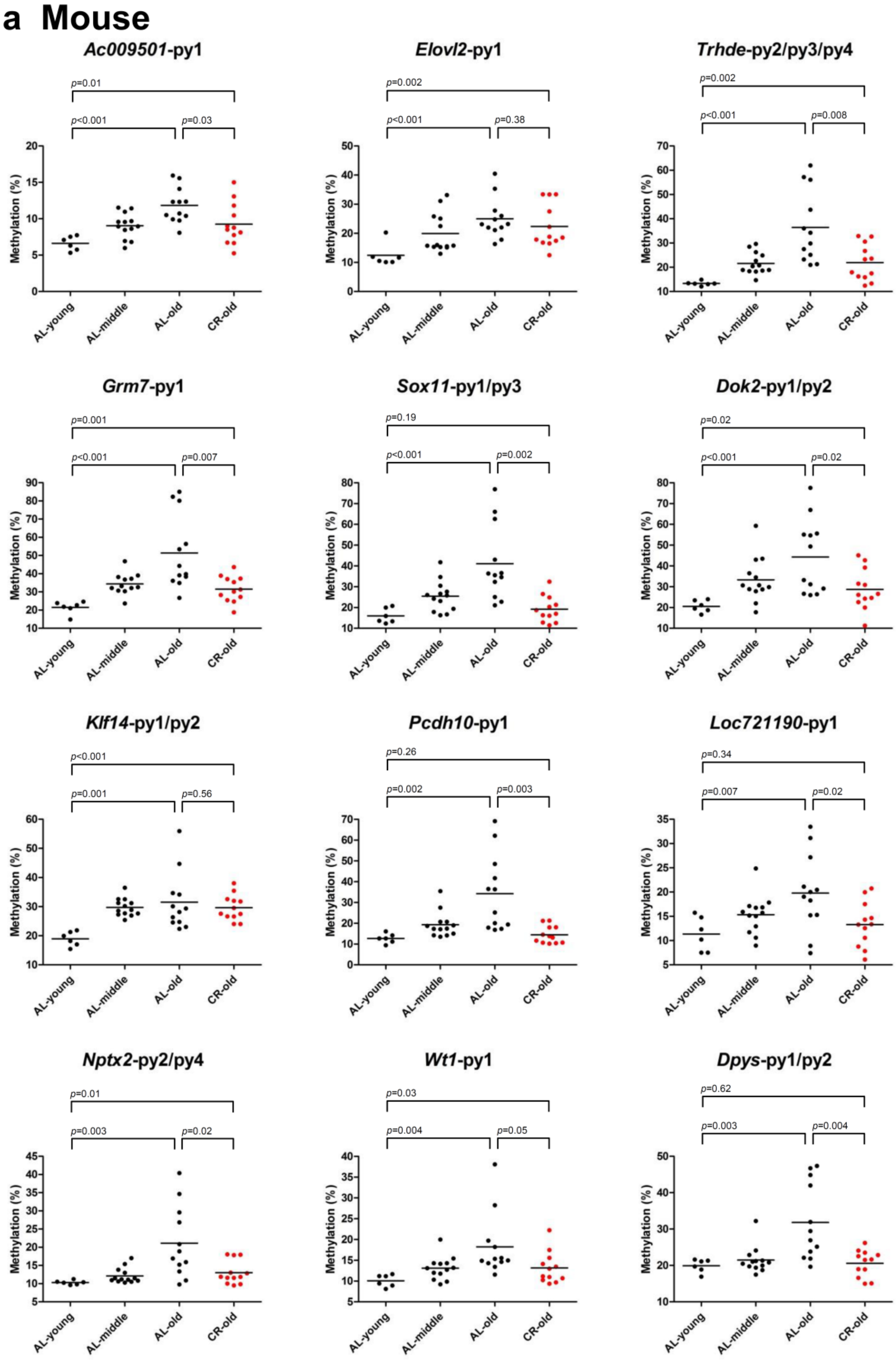
a Mouse
Ac009501-py1
p=0.01
p<0.001
p=0.03
Elovl2-py1
p=0.002
p<0.001
p=0.38
Trhde-py2/py3/py4
p=0.002
p<0.001
p=0.008
Grm7-py1
p=0.001
p<0.001
p=0.007
Sox11-py1/py3
p=0.19
p<0.001
p=0.002
Dok2-py1/py2
p=0.02
p<0.001
p=0.02
Klf14-py1/py2
p<0.001
p=0.001
p=0.56
Pcdh10-py1
p=0.26
p=0.002
p=0.003
Loc721190-py1
p=0.34
p=0.007
p=0.02
Nptx2-py2/py4
p=0.01
p=0.003
p=0.02
Wt1-py1
p=0.03
p=0.004
p=0.05
Dpys-py1/py2
p=0.62
p=0.003
p=0.004
Methylation (%)
AL-young
AL-middle
AL-old
CR-old

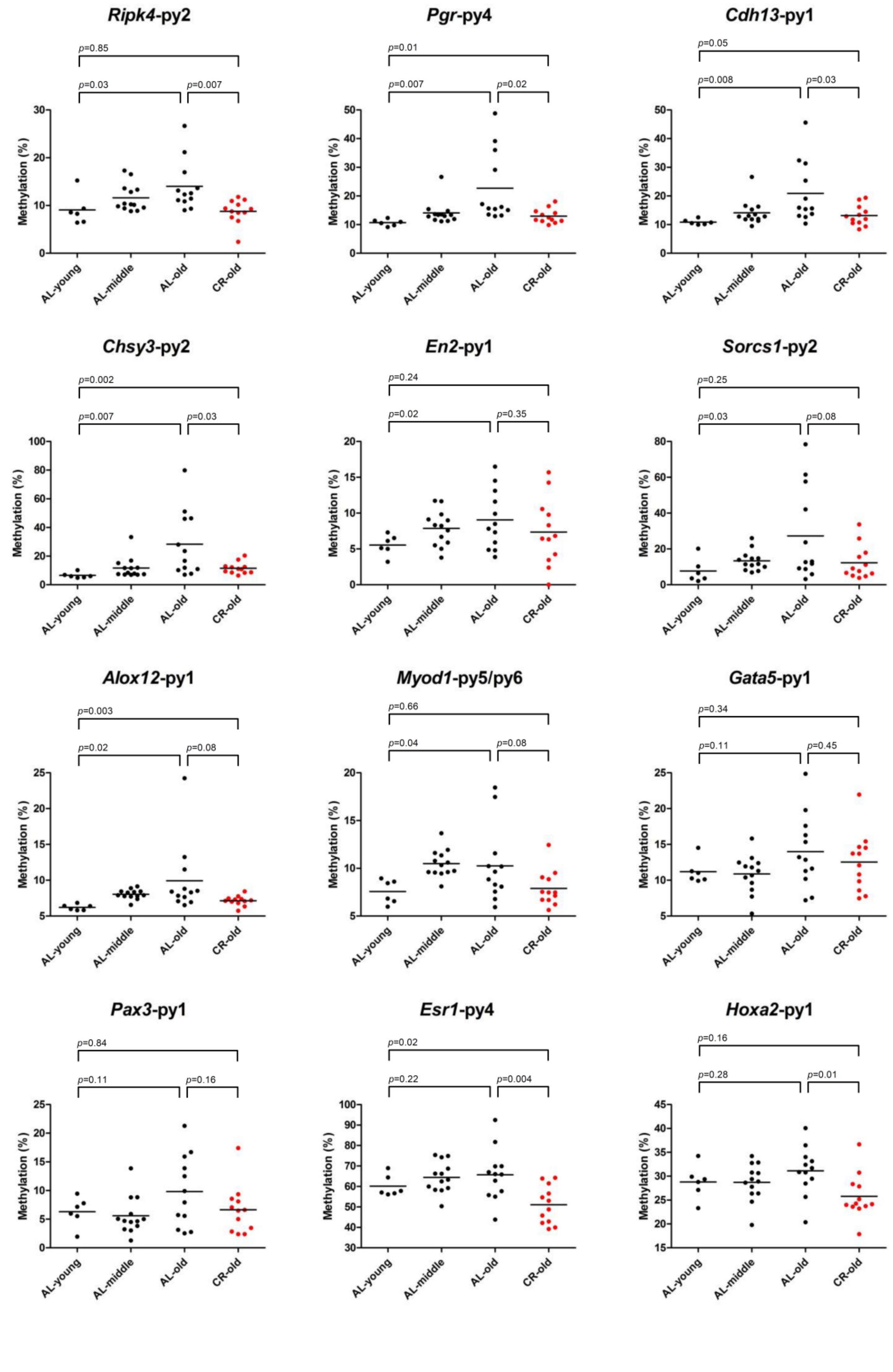

Ripk4-py2
p=0.85
p=0.03
p=0.007
Pgr-py4
p=0.01
p=0.007
p=0.02
Cdh13-py1
p=0.05
p=0.008
p=0.03
Chsy3-py2
p=0.002
p=0.007
p=0.03
En2-py1
p=0.24
p=0.02
p=0.35
Sorcs1-py2
p=0.25
p=0.03
p=0.08
Alox12-py1
p=0.003
p=0.02
p=0.08
Myod1-py5/py6
p=0.66
p=0.04
p=0.08
Gata5-py1
p=0.34
p=0.11
p=0.45
Pax3-py1
p=0.84
p=0.11
p=0.16
Esr1-py4
p=0.02
p=0.22
p=0.004
Hoxa2-py1
p=0.16
p=0.28
p=0.01
Methylation (%)
AL-young
AL-middle
AL-old
CR-old

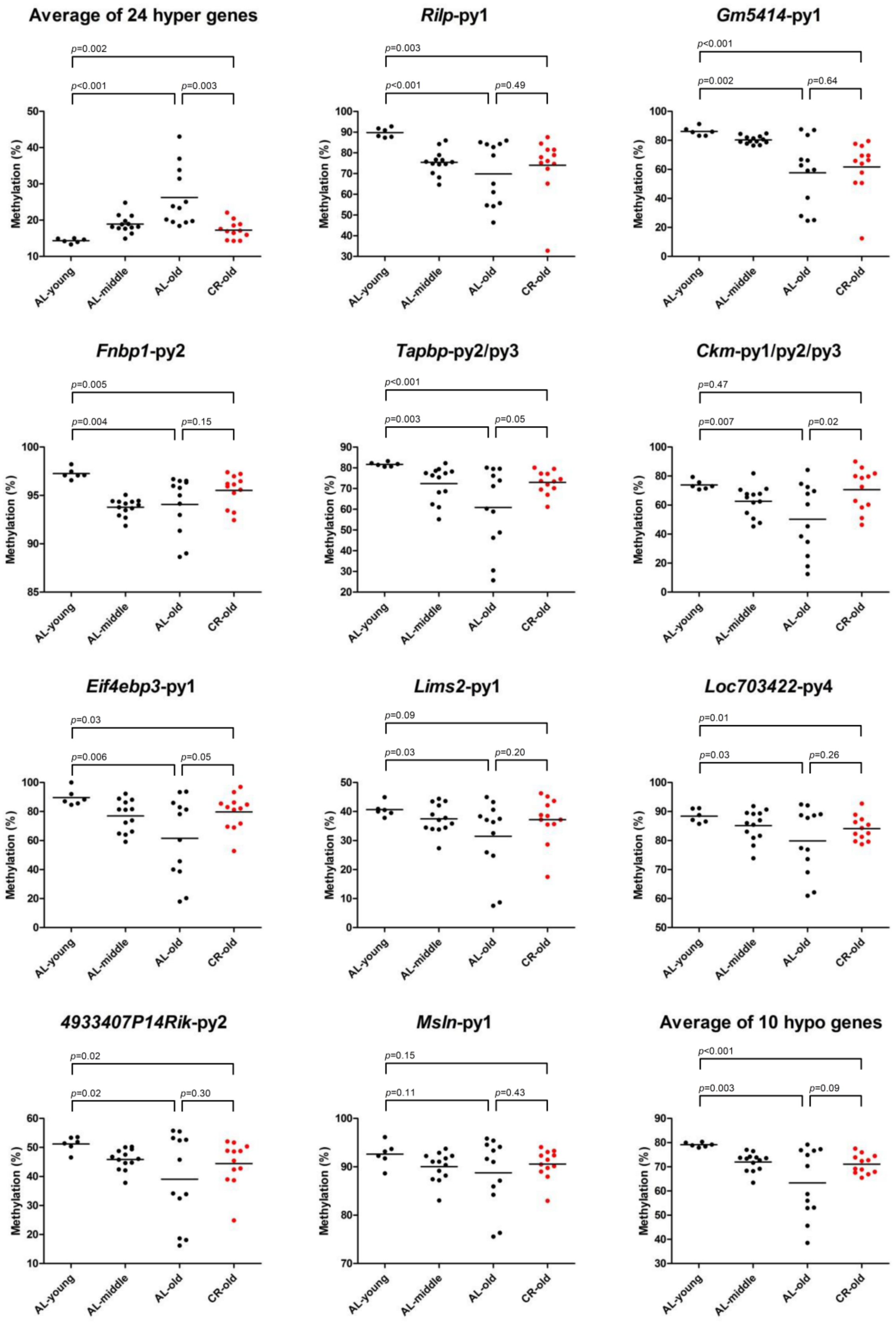
Average of 24 hyper genes
p=0.002
p<0.001
p=0.003
Rilp-py1
p=0.003
p<0.001
p=0.49
Gm5414-py1
p<0.001
p=0.002
p=0.64
Fnbp1-py2
p=0.005
p=0.004
p=0.15
Tapbp-py2/py3
p<0.001
p=0.003
p=0.05
Ckm-py1/py2/py3
p=0.47
p=0.007
p=0.02
Eif4ebp3-py1
p=0.03
p=0.006
p=0.05
Lims2-py1
p=0.09
p=0.03
p=0.20
Loc703422-py4
p=0.01
p=0.03
p=0.26
4933407P14Rik-py2
p=0.02
p=0.02
p=0.30
Msln-py1
p=0.15
p=0.11
p=0.43
Average of 10 hypo genes
p<0.001
p=0.003
p=0.09
Methylation (%)
AL-young
AL-middle
AL-old
CR-old

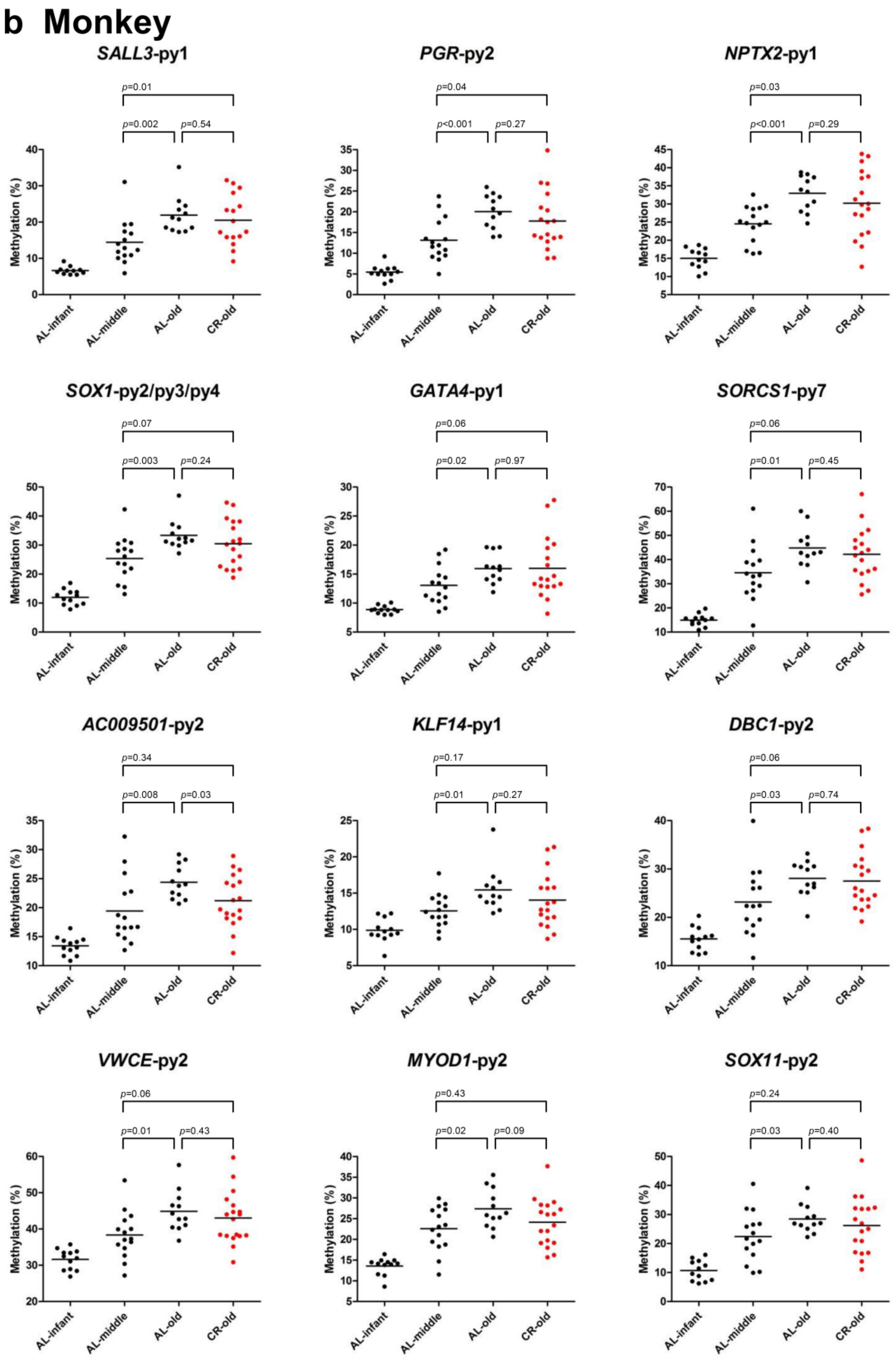
b Monkey
SALL3-py1
p=0.01
p=0.002
p=0.54
PGR-py2
p=0.04
p<0.001
p=0.27
NPTX2-py1
p=0.03
p<0.001
p=0.29
SOX1-py2/py3/py4
p=0.07
p=0.003
p=0.24
GATA4-py1
p=0.06
p=0.02
p=0.97
SORCS1-py7
p=0.06
p=0.01
p=0.45
AC009501-py2
p=0.34
p=0.008
p=0.03
KLF14-py1
p=0.17
p=0.01
p=0.27
DBC1-py2
p=0.06
p=0.03
p=0.74
VWCE-py2
p=0.06
p=0.01
p=0.43
MYOD1-py2
p=0.43
p=0.02
p=0.09
SOX11-py2
p=0.24
p=0.03
p=0.40
Methylation (%)
AL-infant
AL-middle
AL-old
CR-old

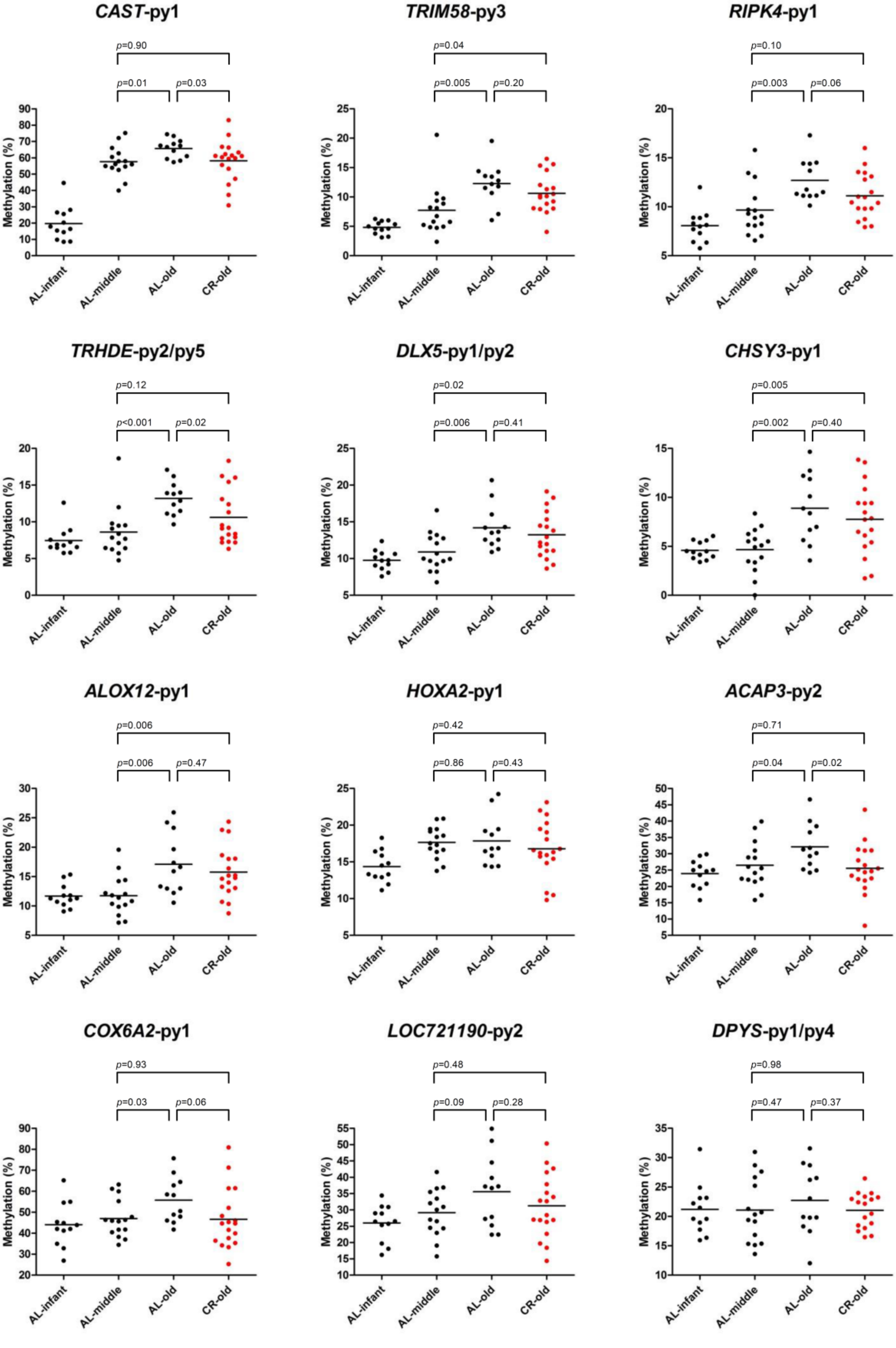

CAST-py1
p=0.90
p=0.01
p=0.03
TRIM58-py3
p=0.04
p=0.005
p=0.20
RIPK4-py1
p=0.10
p=0.003
p=0.06
TRHDE-py2/py5
p=0.12
p<0.001
p=0.02
DLX5-py1/py2
p=0.02
p=0.006
p=0.41
CHSY3-py1
p=0.005
p=0.002
p=0.40
ALOX12-py1
p=0.006
p=0.006
p=0.47
HOXA2-py1
p=0.42
p=0.86
p=0.43
ACAP3-py2
p=0.71
p=0.04
p=0.02
COX6A2-py1
p=0.93
p=0.03
p=0.06
LOC721190-py2
p=0.48
p=0.09
p=0.28
DPYS-py1/py4
p=0.98
p=0.47
p=0.37
Methylation (%)
AL-infant
AL-middle
AL-old
CR-old

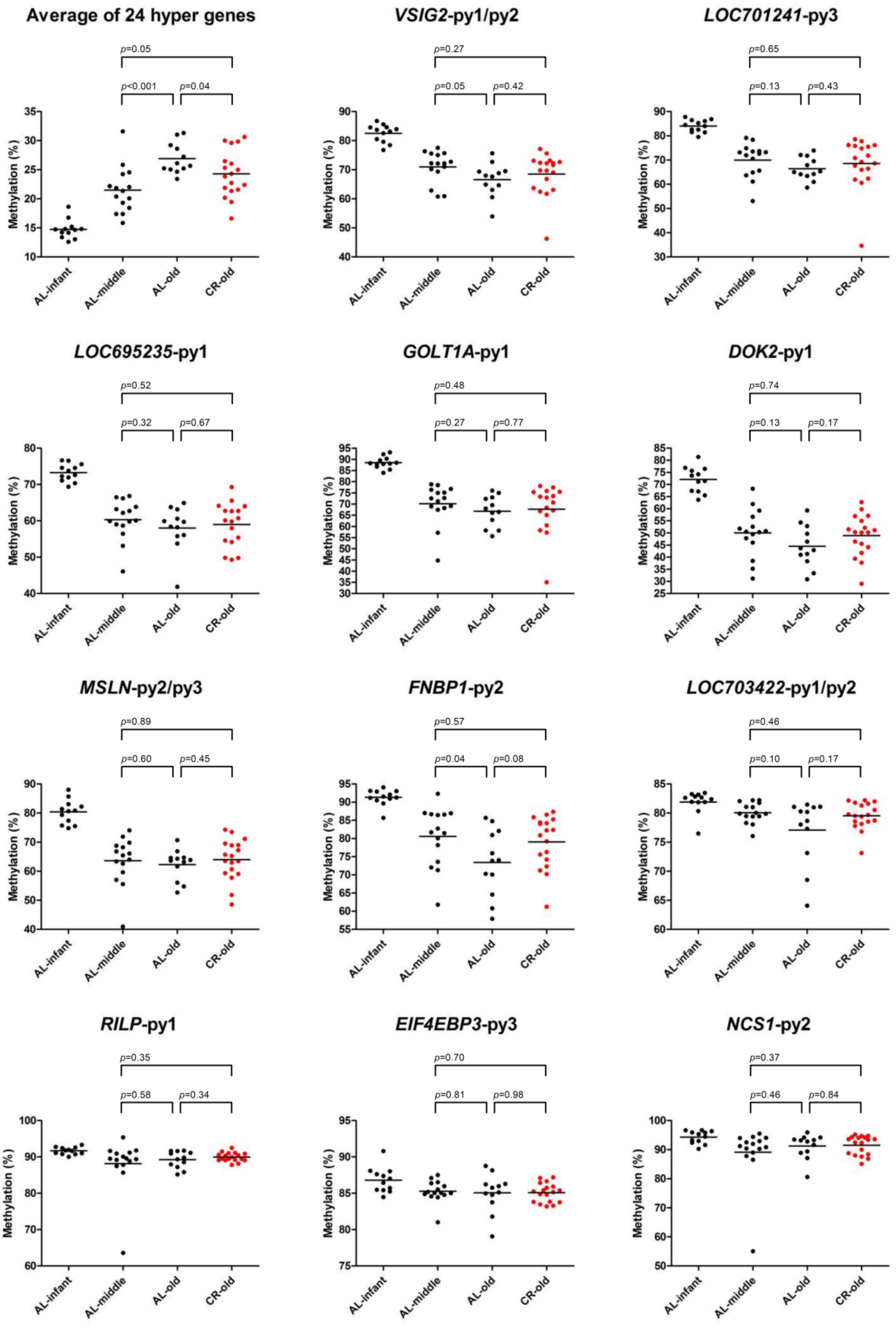
Average of 24 hyper genes
p=0.05
p<0.001
p=0.04
VSIG2-py1/py2
p=0.27
p=0.05
p=0.42
LOC701241-py3
p=0.65
p=0.13
p=0.43
LOC695235-py1
p=0.52
p=0.32
p=0.67
GOLT1A-py1
p=0.48
p=0.27
p=0.77
DOK2-py1
p=0.74
p=0.13
p=0.17
MSLN-py2/py3
p=0.89
p=0.60
p=0.45
FNBP1-py2
p=0.57
p=0.04
p=0.08
LOC703422-py1/py2
p=0.46
p=0.10
p=0.17
RILP-py1
p=0.35
p=0.58
p=0.34
EIF4EBP3-py3
p=0.70
p=0.81
p=0.98
NCS1-py2
p=0.37
p=0.46
p=0.84
Methylation (%)
AL-infant
AL-middle
AL-old
CR-old

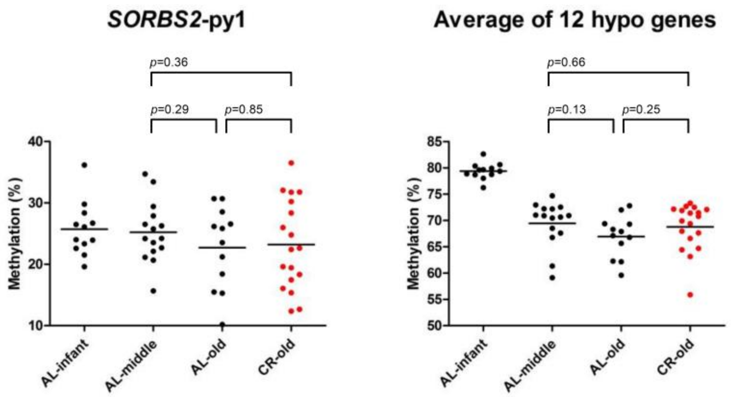

Supplementary Figure 12 | CR effects on aging drift detected by bisulfite pyrosequencing assay. DNA methylation profiles in AL and CR animals (**a**; mice, and **b**; monkeys). The averaged data were derived from the methylation data of hypermethylated genes (n=24 in mice, n=24 in monkeys) and hypomethylated genes (n=10 in mice, n=12 in monkeys) listed in Supplementary Tables 14 and 15. Each dot corresponds to one individual (mouse; AL-young, n=6, AL-middle, n=13, AL-old, n=12, CR-old, n=12, monkey; AL-infant, n=12, AL-middle, n=15, AL-old, n=12, CR-old, n=18). The bar in the graphs represents the median. *p*-values were obtained using the unpaired t-test with Welch's correction.

Mouse

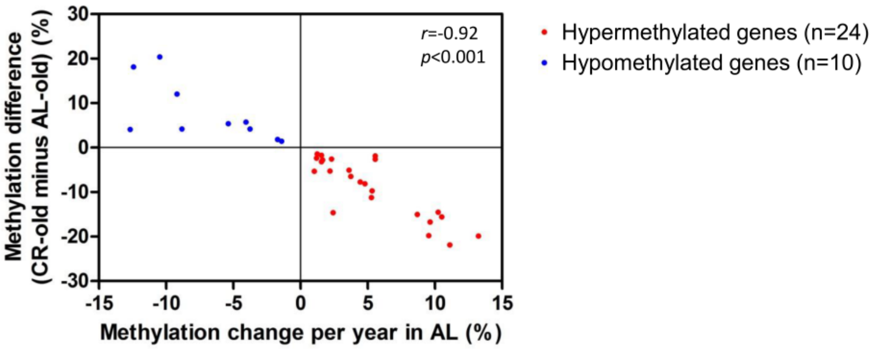

Monkey

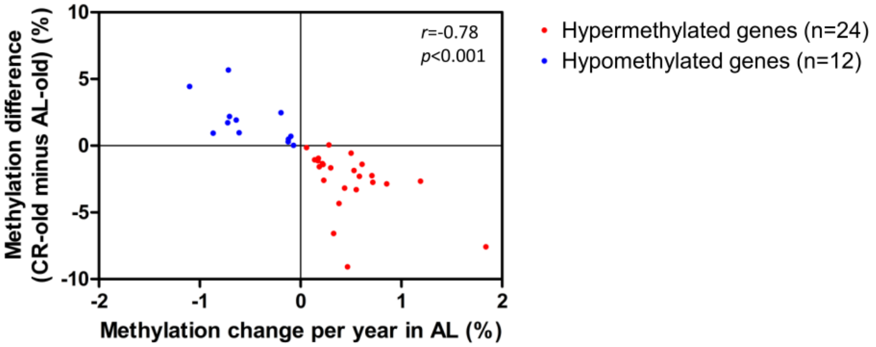

Supplementary Figure 13 | Correlation between changes in methylation by CR and changes in methylation by age. The x-axis is methylation changes per year in AL fed animals. Positive/negative value means methylation increases/decreases with age, respectively. The y-axis is the differences of methylation percentage between CR-old and AL-old animals. Each dot represents an averaged value of DNA methylation status detected by each bisulfite pyrosequencing assay. Spearman's rank correlation coefficient (*r*) and corresponding two-tailed *p*-value were calculated by GraphPad.

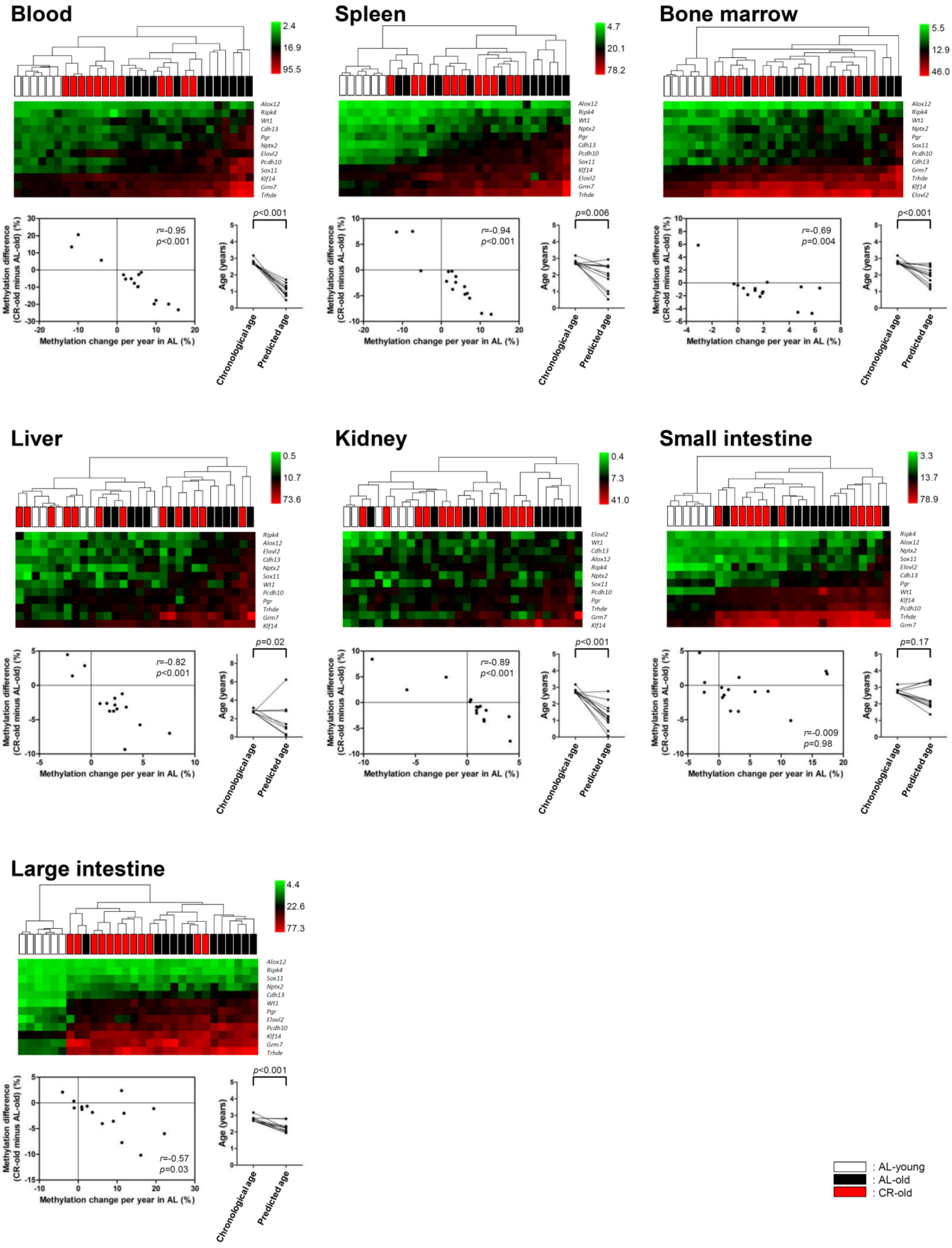

Blood
r=-0.95
p<0.001
Spleen
r=-0.94
p<0.001
p=0.006
Bone marrow
r=-0.69
p=0.004
Liver
r=-0.82
p<0.001
p=0.02
Kidney
r=-0.89
p<0.001
Small intestine
r=-0.009
p=0.98
p=0.17
Large intestine
r=-0.57
p=0.03
p<0.001
Methylation difference (CR-old minus AL-old) (%)
Methylation change per year in AL (%)
Age (years)
Chronological age
Predicted age
: AL-young
: AL-old
: CR-old

Supplementary Figure 14 | CR effects on aging drift detected by bisulfite pyrosequencing assay in multiple tissues. Unsupervised hierarchical clustering analysis of the 12 loci assayed. The green to red color scale indicates the methylation percentage. The color codes of caloric status and age are shown on the bottom-right. Correlation between changes in methylation by CR and changes in methylation by age are shown in the lower-left of the clustering. The x-axis is methylation changes per year in AL fed animals. Positive/negative value means methylation increases/decreases with age, respectively. The y-axis is the differences of methylation percentage between CR-old and AL-old animals. Each dot represents an average value of DNA methylation status detected by each bisulfite pyrosequencing assay. Spearman's rank correlation coefficient (*r*) and corresponding two-tailed *p*-value were calculated by GraphPad. Differences between the predicted ages and chronological ages in CR-old animals are shown in the lower-right of the clustering. Each pair of dots connected by a line represents the difference between predicted age and chronological age in each individual. Paired t-test was used for *p*-value calculation.

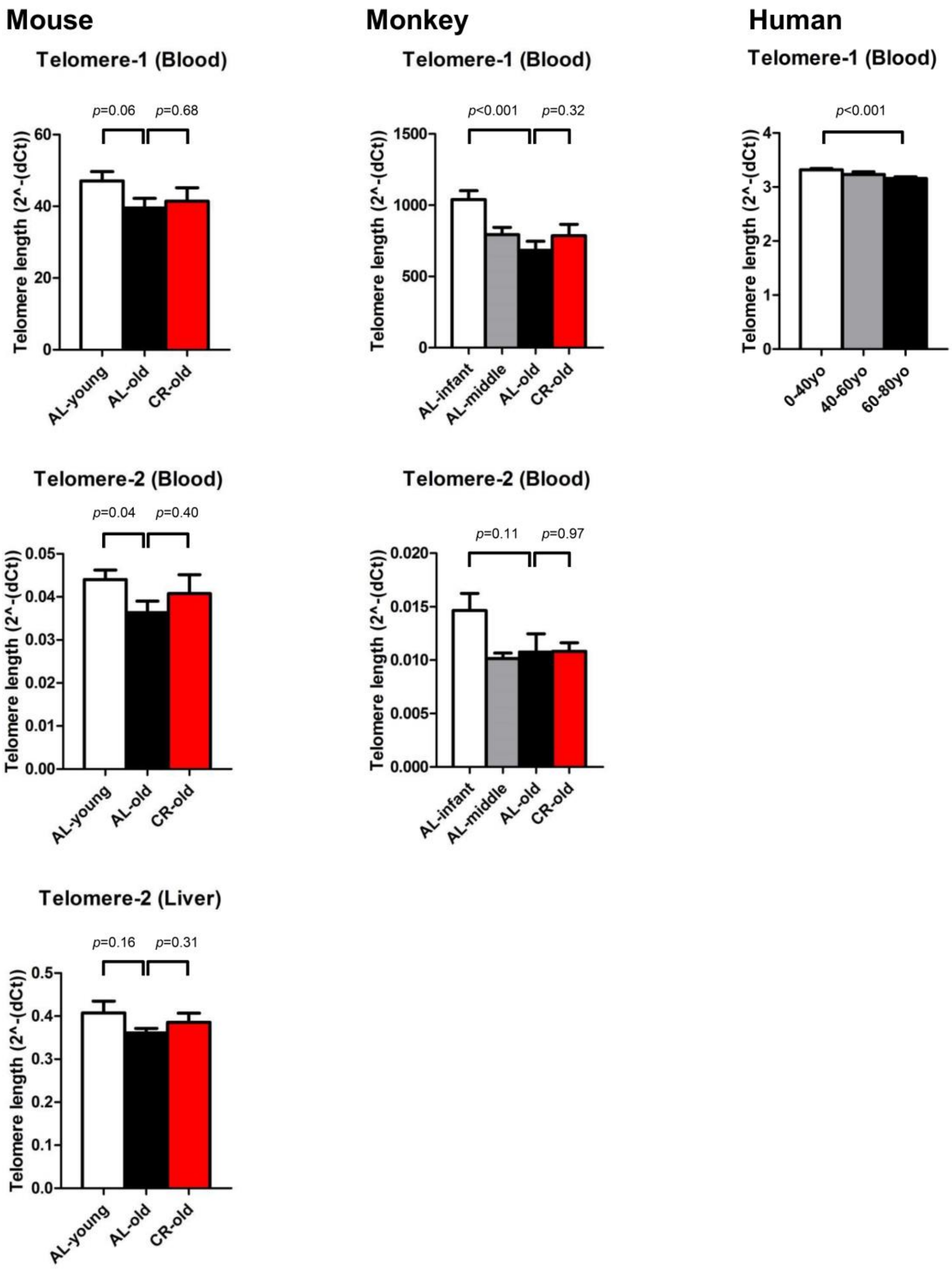

Supplementary Figure 15 | Correlation between telomere length and age. Relative telomere length was shown in mice, monkeys and humans. Relative telomere length declined with age in AL animals. There was no statistical evidence of a CR effect in telomere shortening. Two primer sets were used to amplify telomeres by quantitative PCR. The values are presented as means of triplicate determinations (PCR reactions). Bars represent standard error. *p*-values were obtained using the unpaired t-test with Welch's correction.

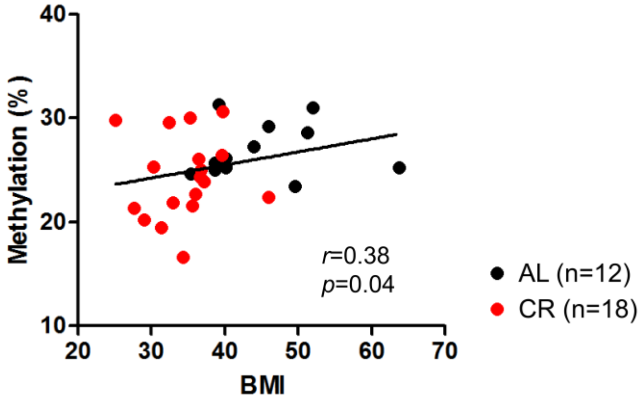

Supplementary Figure 16 | Correlation between DNA methylation and BMI in monkey. The association between Body Mass Index (BMI) (x-axis) and average methylation percentage of 24 hypermethylated ARM genes (y-axis). Each dot corresponds to one individual. Black and red dots represent AL and CR animals, respectively. Correlation between methylation and BMI was calculated using Spearman's correlation (*r*). *r*-values and the corresponding two-tailed *p*-values were calculated by GraphPad.

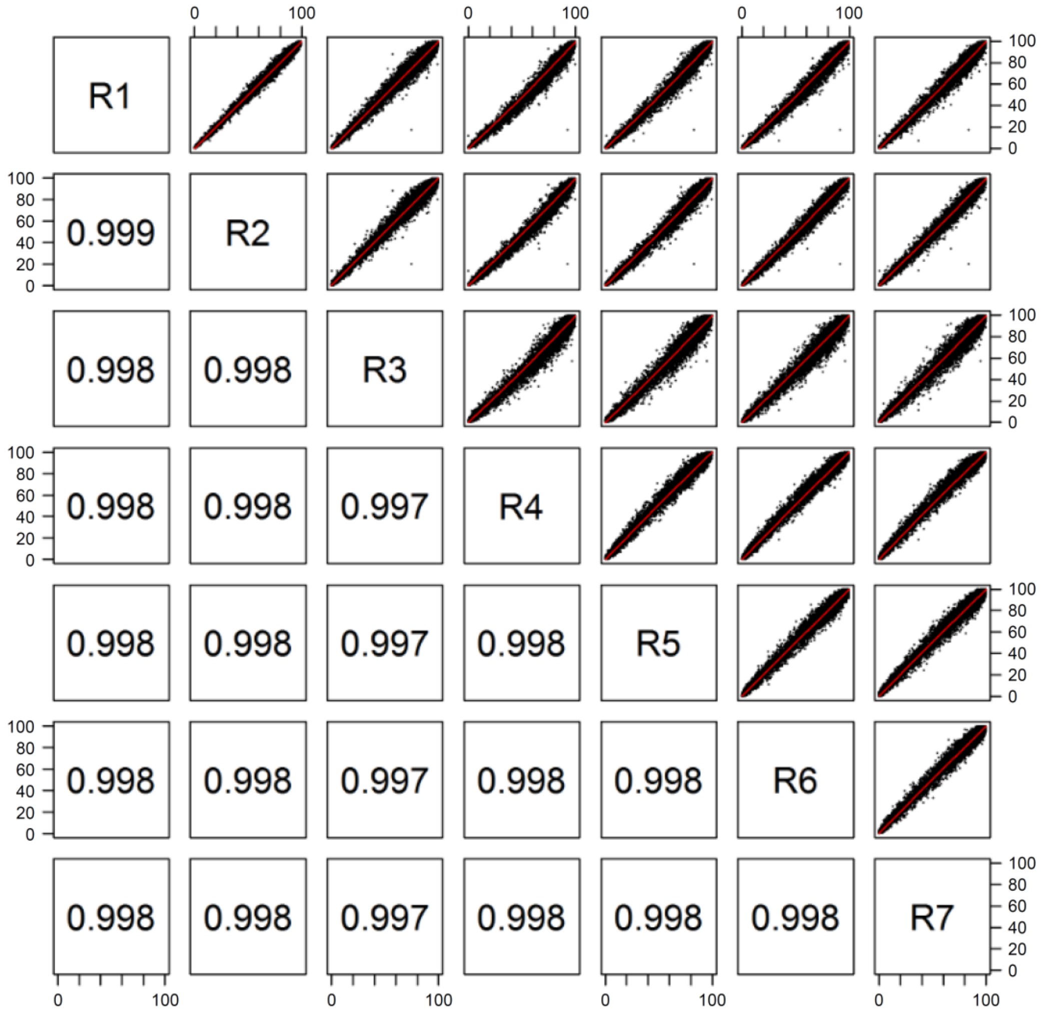

Supplementary Figure 17 | Seven technical replicates of human whole blood sample in DREAM analysis. Correlation matrix of Pearson *r* and scatter plots of DNA methylation levels in all genomic regions (sequencing depth ≥100 reads, 25,780 sites). The lowess regression line is shown in red. Correlations are significant at $p<0.001$ (two-tailed).

Supplementary Table 1 | Samples used.

**Whole blood**	All samples		Samples for DREAM (Subset of all samples)	
	N	Age (years) Median (range)	N	Age (years) Median (range)
**Mouse (C57BL/6)**	**Sample set A**		**Sample set D**	
Young (AL):	6	0.3 (0.3)	5	0.3 (0.3)
Middle (AL):	13	1.1 (1.1-1.6)	7	1.2 (1.1-1.6)
Old (AL):	12	2.5 (2.4-2.8)	7	2.5 (2.4-2.8)
Old (40% CR):	12	2.7 (2.7-3.2)	5	2.8 (2.7-3.2)
**Monkey**	**Sample set B**		**Sample set E**	
Infant (AL):	12	0.9 (0.8-0.9)	4	0.9 (0.8-0.9)
Middle (AL):	15	11.4 (10-13)	7	11.4 (10-12)
Old (AL):	12	25.9 (21-30)	5	26.5 (22-30)
Old (30% CR):	18	27.8 (22-30)	6	26.0 (24-30)
**Human**	**Sample set C**		**Sample set F**	
Newborn:	13	0.0 (0)	5	0.0 (0)
Young:	54	23.7 (0-40)		
Middle:	27	49.6 (40-60)	5	22.0 (21-55)
Old:	45	69.6 (60-86)	6	79.0 (70-86)

**PBMC (peripheral blood mononuclear cell)**	
**Monkey**	**Sample set G**
4 monkeys	(age interval: 4-5 years)

**Multiple tissues (Spl, BM, Lvr, Kid, SI, LI)**		
	N	Age (years) Median (range)
**Mouse (C57BL/6)**	**Sample set H**	
Young (AL):	6	0.3 (0.3)
Old (AL):	12	2.5 (2.4-2.8)
Old (40% CR):	12	2.7 (2.7-3.2)

N; Number of samples
AL; ad libitum
CR; Caloric restriction
Spl; Spleen
BM; Bone marrow
Lvr; Liver
kid; Kidney
SI; Small intestine
LI; Large intestine

Supplementary Table 2 | Summary of sample sets and assays.

Species	Tissue	Sample set and assay	Number of assays

		DNA methylation (DREAM)
Mouse	Whole blood	Sample set D
Monkey	Whole blood	Sample set E
Human	Whole blood	Sample set F

		DNA methylation (PSQ)	PSQ (Hyper-met / Hypo-met)
Mouse	Whole blood	Sample set A	24 / 10
Monkey	Whole blood	Sample set B	24 / 12
Human	Whole blood	Sample set C	14 / 2

		DNA methylation (PSQ)	PSQ (Hyper-met / Hypo-met)
Monkey	PBMC	Sample set G	2 / 1

		DNA methylation (PSQ)	PSQ (Hyper-met / Hypo-met)
Mouse	Whole blood	Sample set A	12 / 3
Mouse	Spleen	Sample set H	
Mouse	Bone marrow	Sample set H	
Mouse	Liver	Sample set H	
Mouse	Kidney	Sample set H	
Mouse	Small intestine	Sample set H	
Mouse	Large intestine	Sample set H	

		Gene expression (TaqMan)	TaqMan (Hyper-met / Hypo-met)
Mouse	Liver	Sample set H	2 / 2

		Telomere length (RT-PCR)
Mouse	Whole blood	Sample set A
Mouse	Liver	Sample set H
Monkey	Whole blood	Sample set B
Human	Whole blood	Sample set C

Sample set; defined in Supplementary Table 1
PSQ; Bisulfite Pyrosequencing
PBMC; peripheral blood mononuclear cell
Hyper-met; Age-related hypermethylated gene
Hypo-met; Age-related hypomethylated gene

Supplementary Table 3 | Pearson correlation matrix between cord blood samples by methylation status detected by 16 pyrosequencing assays.

	CB_10300	CB_9442	CB_9416	CB_9677	CB_10214	CB_10293	CB_10265	CB_9952	CB_139941	CB_9576	CB_9880	CB_9671	CB_9524
CB_10300	1												
CB_9442	0.99	1											
CB_9416	0.99	0.98	1										
CB_9677	0.98	0.99	0.97	1									
CB_10214	0.98	0.96	0.99	0.95	1								
CB_10293	0.99	0.98	0.98	0.97	0.98	1							
CB_10265	1.00	0.99	0.98	0.98	0.98	0.99	1						
CB_9952	0.99	0.98	0.97	0.97	0.96	0.98	0.99	1					
CB_139941	0.97	0.99	0.96	0.97	0.95	0.98	0.98	0.95	1				
CB_9576	0.99	1.00	0.98	0.99	0.96	0.98	0.99	0.98	0.98	1			
CB_9880	0.99	0.99	0.97	0.98	0.95	0.99	0.99	0.97	0.99	0.99	1		
CB_9671	1.00	0.99	0.98	0.98	0.97	0.99	1.00	0.99	0.98	0.99	0.99	1	
CB_9524	0.98	0.99	0.97	0.98	0.95	0.98	0.99	0.98	0.98	0.99	0.99	0.99	1

Correlations are significant at $p<0.001$ (2-tailed).

Supplementary Table 4 | Power and sample size calculations for methylation difference between young and old samples.

Mouse

Region of sites	p1	p2	Sample size required (minimal coverage at each site) alpha=0.000001, power=0.8	Sample size required (minimal coverage at each site) alpha=0.0001, power=0.5	Sample size analyzed (reads/group/site)	Power alpha=0.000001	Power alpha=0.0001
All sites	0.58	0.51	3325	1533	4373	0.95	1.00
CGI	0.12	0.25	585	271	9326	1.00	1.00
CGI (Y<5%)	0.02	0.18	229	107	3894	1.00	1.00
nCGI	0.68	0.56	1074	496	3291	1.00	1.00
nCGI (Y>90%)	0.94	0.78	307	143	2825	1.00	1.00

Monkey

Region of sites	p1	p2	Sample size required (minimal coverage on each site) alpha=0.000001, power=0.8	Sample size required (minimal coverage on each site) alpha=0.0001, power=0.5	Sample size analyzed (average coverage per site)	Power alpha=0.000001	Power alpha=0.0001
All sites	0.76	0.58	447	207	3478	1.00	1.00
CGI	0.44	0.49	6540	3013	3058	0.17	0.51
CGI (I<5%)	0.02	0.22	172	80	2263	1.00	1.00
nCGI	0.78	0.60	432	200	3514	1.00	1.00
nCGI (I>90%)	0.94	0.73	203	95	3527	1.00	1.00

Human

Region of sites	p1	p2	Sample size required (minimal coverage on each site) alpha=0.000001, power=0.8	Sample size required (minimal coverage on each site) alpha=0.0001, power=0.5	Sample size analyzed (average coverage per site)	Power alpha=0.000001	Power alpha=0.0001
All sites	0.58	0.51	3325	1533	7333	1.00	1.00
CGI	0.47	0.51	10266	4729	14787	0.98	1.00
CGI (N<5%)	0.03	0.20	230	107	6967	1.00	1.00
nCGI	0.61	0.52	1993	919	5437	1.00	1.00
nCGI (N>90%)	0.93	0.74	249	116	3820	1.00	1.00

Sites analyzed; standard deviation>10%
Y; Average methylation % in young
I; Average methylation % in infant
N; Average methylation % in newborn
p1; Average methylation ratio in young/infant/newborn
p2; Average methylation ratio in old
Calculation for specified Sample Size
alpha=0.000001 or 0.0001
power=0.8 or 0.5
2 Sided Test
Calculation for specified Power
alpha=0.000001 or 0.0001
2 Sided Test

Supplementary Table 5 | Number of sites showing methylation drifts with different threshold.

Mouse DREAM

Ave Met of all ≥ 1%	Real sites 8728	%	Permuted sites 8728000	%	Obs/Exp	Chi-test
r≤ -0.4	2189	25.1	853910	9.8	2.6	*p*<0.001
r≥ 0.4	1774	20.3	837379	9.6	2.1	*p*<0.001
***r*≤ -0.5**	**1521**	**17.4**	**445065**	**5.1**	**3.4**	***p*<0.001**
***r*≥ 0.5**	**1341**	**15.4**	**430081**	**4.9**	**3.1**	***p*<0.001**
r≤ -0.6	941	10.8	197549	2.3	4.8	*p*<0.001
r≥ 0.6	940	10.8	187318	2.1	5.0	*p*<0.001

Monkey DREAM

Ave Met of all ≥ 1%	Real sites 19045	%	Permuted sites 19044000	%	Obs/Exp	Chi-test
r≤ -0.4	7482	39.3	1218914	6.4	6.1	*p*<0.001
r≥ 0.4	5010	26.3	1212908	6.4	4.1	*p*<0.001
***r*≤ -0.5**	**6022**	**31.6**	**492451**	**2.6**	**12.2**	***p*<0.001**
***r*≥ 0.5**	**4462**	**23.4**	**486515**	**2.6**	**9.2**	***p*<0.001**
r≤ -0.6	4364	22.9	156657	0.8	27.9	*p*<0.001
r≥ 0.6	3796	19.9	151940	0.8	25.0	*p*<0.001

Human DREAM

Ave Met of all ≥ 1%	Real sites 20682	%	Permuted sites 20682000	%	Obs/Exp	Chi-test
r≤ -0.4	5997	29.0	1525281	7.4	3.9	*p*<0.001
r≥ 0.4	3618	17.5	1468771	7.1	2.5	*p*<0.001
***r*≤ -0.5**	**4244**	**20.5**	**673649**	**3.3**	**6.3**	***p*<0.001**
***r*≥ 0.5**	**2575**	**12.5**	**635131**	**3.1**	**4.1**	***p*<0.001**
r≤ -0.6	2766	13.4	238355	1.2	11.6	*p*<0.001
r≥ 0.6	1560	7.5	218962	1.1	7.1	*p*<0.001

Supplementary Table 6 | Significant terms in Ingenuity Pathway Analysis of age-related genes in AL-mice.

Top Canonical Pathways

Name	*p*-value	Ratio
Axonal Guidance Signaling	1.26E-06	24/433 (0.055)
Molecular Mechanisms of Cancer	3.85E-05	19/365 (0.052)
Factors Promoting Cardiogenesis in Vertebrates	4.22E-05	9/92 (0.098)
Renin-Angiotensin Signaling	1.58E-04	9/109 (0.083)
Chondroitin Sulfate Biosynthesis (Late Stages)	1.67E-04	6/46 (0.13)

Top Upstream Regulators

Upstream Regulator	*p*-value of overlap
TGFB1	1.75E-07
POU4F1	2.60E-07
beta-estradiol	8.39E-07
SOX2	1.75E-06
SHH	3.74E-06

Top Diseases and Bio Functions

Diseases and Disorders

Name	*p*-value	# Molecules
Cancer	6.38E-16 - 1.75E-03	335
Gastrointestinal Disease	1.52E-14 - 3.90E-04	258
Organismal Injury and Abnormalities	2.05E-14 - 1.42E-03	190
Reproductive System Disease	2.05E-14 - 9.51E-04	163
Hepatic System Disease	4.96E-09 - 4.96E-09	164

Molecular and Cellular Functions

Name	*p*-value	# Molecules
Cellular Development	3.22E-16 - 1.82E-03	152
Cellular Assembly and Organization	7.91E-12 - 1.62E-03	96
Cellular Function and Maintenance	7.91E-12 - 1.62E-03	88
Cell Morphology	2.91E-11 - 1.40E-03	118
Cellular Growth and Proliferation	4.39E-11 - 1.51E-03	147

Physiological System Development and Function

Name	*p*-value	# Molecules
Embryonic Development	6.48E-21 - 1.67E-03	145
Organismal Development	6.48E-21 - 1.67E-03	165
Organ Development	4.76E-14 - 1.67E-03	126
Renal and Urological System Development and Function	4.76E-14 - 1.70E-03	57

Reproductive System Development and Function	4.76E-14 - 1.51E-03	48

Top Tox Functions

Assays: Clinical Chemistry and Hematology

Name	*p*-value	# Molecules
Increased Levels of Alkaline Phosphatase	3.59E-02 - 3.59E-02	4
Increased Levels of Red Blood Cells	7.01E-02 - 5.35E-01	2
Increased Levels of LDH	2.39E-01 - 2.39E-01	1
Increased Levels of Creatinine	4.99E-01 - 4.99E-01	1

Cardiotoxicity

Name	*p*-value	# Molecules
Cardiac Hypoplasia	4.57E-05 - 4.90E-01	4
Congenital Heart Anomaly	4.49E-04 - 1.03E-01	11
Cardiac Arrythmia	5.60E-03 - 4.48E-01	10
Cardiac Hemorrhaging	6.40E-03 - 5.31E-02	2
Cardiac Hypertrophy	6.92E-03 - 4.24E-01	12

Hepatotoxicity

Name	*p*-value	# Molecules
Liver Hyperplasia/Hyperproliferation	4.96E-09 - 1.03E-01	165
Liver Adhesion	3.04E-04 - 1.03E-01	3
Hepatocellular Carcinoma	5.26E-03 - 9.94E-02	17
Liver Cholestasis	1.80E-02 - 2.88E-01	2
Liver Cirrhosis	3.57E-02 - 5.18E-01	3

Nephrotoxicity

Name	*p*-value	# Molecules
Renal Dysplasia	1.29E-04 - 7.01E-02	5
Renal Hypoplasia	2.73E-03 - 7.01E-02	4
Renal Hyperplasia/Hyperproliferation	6.06E-03 - 4.97E-02	3
Glomerular Injury	1.80E-02 - 3.17E-01	8
Kidney Failure	1.80E-02 - 1.29E-01	7

Top Networks

Associated Network Functions	Score
1 Embryonic Development, Organismal Development, Skeletal and Muscular System Development and Function	48
2 Cell Morphology, Cellular Assembly and Organization, Cellular Development	45
3 Embryonic Development, Tissue Morphology, Organismal Development	43
4 Developmental Disorder, Hereditary Disorder,	41

Ophthalmic Disease		
5 Nervous System Development and Function, Cellular Movement, Cell-To-Cell Signaling and Interaction	39	

Top Tox Lists

Name	*p*-value	Ratio
Increases Glomerular Injury	2.20E-03	5/51 (0.098)
PPARa/RXRa Activation	1.81E-02	8/183 (0.044)
Hepatic Fibrosis	3.34E-02	5/99 (0.051)
Biogenesis of Mitochondria	4.97E-02	2/20 (0.1)
Increases Cardiac Proliferation	4.98E-02	3/46 (0.065)

Supplementary Table 7 | Significant terms in Ingenuity Pathway Analysis of age-related genes in AL-monkeys.

Top Canonical Pathways

Name	*p*-value	Ratio
Transcriptional Regulatory Network in Embryonic Stem Cells	7.79E-06	8/40 (0.2)
Cellular Effects of Sildenafil (Viagra)	1.51E-04	12/129 (0.093)
Gai Signaling	3.21E-04	11/120 (0.092)
Corticotropin Releasing Hormone Signaling	6.82E-04	10/111 (0.09)
Role of NANOG in Mammalian Embryonic Stem Cell Pluripotency	6.82E-04	10/111 (0.09)

Top Upstream Regulators

Upstream Regulator	*p*-value of overlap
CTNNB1	8.39E-12
PITX2	2.98E-10
beta-estradiol	2.30E-08
LMX1B	7.03E-08
SHH	4.42E-07

Top Diseases and Bio Functions

Diseases and Disorders

Name	*p*-value	# Molecules
Cancer	4.07E-26 - 6.03E-04	498
Gastrointestinal Disease	7.95E-14 - 6.62E-04	368
Organismal Injury and Abnormalities	2.62E-12 - 6.87E-04	280
Reproductive System Disease	2.62E-12 - 5.31E-04	211
Hepatic System Disease	3.12E-12 - 4.97E-12	239

Molecular and Cellular Functions

Name	*p*-value	# Molecules
Cellular Development	7.60E-13 - 6.35E-04	188
Cellular Movement	6.98E-11 - 5.49E-04	144
Cell-To-Cell Signaling and Interaction	1.57E-10 - 6.87E-04	105
Molecular Transport	1.94E-09 - 6.35E-04	137
Cell Morphology	2.55E-09 - 5.80E-04	166

Physiological System Development and Function

Name	*p*-value	# Molecules
Behavior	2.14E-18 - 6.09E-04	110
Embryonic Development	4.15E-16 - 6.87E-04	173
Organismal Development	4.15E-16 - 6.87E-04	231
Nervous System Development and Function	1.06E-14 - 6.25E-04	186
Organismal Survival	1.12E-14 - 2.83E-04	163

Top Tox Functions

Assays: Clinical Chemistry and Hematology

Name	*p*-value	# Molecules
Increased Levels of Alkaline Phosphatase	3.47E-02 - 3.47E-02	5

Increased Levels of Red Blood Cells	4.60E-02 - 4.60E-02	6
Increased Levels of Albumin	5.18E-02 - 1.92E-01	2
Decreased Levels of Albumin	1.24E-01 - 1.24E-01	1
Increased Levels of ALT	1.47E-01 - 1.47E-01	1

Cardiotoxicity

Name	*p*-value	# Molecules
Congenital Heart Anomaly	3.59E-05 - 1.47E-01	17
Heart Failure	4.21E-04 - 1.92E-01	18
Cardiac Dysfunction	2.79E-03 - 1.92E-01	7
Cardiac Hypertrophy	9.23E-03 - 1.44E-01	19
Cardiac Enlargement	1.32E-02 - 1.47E-01	3

Hepatotoxicity

Name	*p*-value	# Molecules
Liver Hyperplasia/Hyperproliferation	3.12E-12 - 5.12E-01	239
Liver Hepatomegaly	4.67E-03 - 4.67E-03	7
Glutathione Depletion In Liver	2.62E-02 - 3.29E-01	2
Liver Cholestasis	2.62E-02 - 4.61E-01	4
Liver Cirrhosis	2.62E-02 - 3.44E-01	6

Nephrotoxicity

Name	*p*-value	# Molecules
Renal Inflammation	6.87E-04 - 4.99E-01	9
Renal Nephritis	6.87E-04 - 4.99E-01	9
Glomerular Injury	1.08E-03 - 2.54E-01	11
Renal Fibrosis	1.08E-03 - 1.37E-01	8
Renal Hypoplasia	1.89E-03 - 2.62E-02	6

Top Networks

Associated Network Functions	Score
1 Cellular Development, Tissue Development, Nervous System Development and Function	41
2 Embryonic Development, Organismal Development, Cellular Development	41
3 Connective Tissue Disorders, Developmental Disorder, Hereditary Disorder	37
4 Molecular Transport, Cell Death and Survival, Cell-To-Cell Signaling and Interaction	37
5 Cardiovascular Disease, Organismal Injury and Abnormalities, Nucleic Acid Metabolism	32

Top Tox Lists

Name	*p*-value	Ratio
Hepatic Fibrosis	4.51E-03	8/99 (0.081)
Cardiac Hypertrophy	7.33E-03	19/389 (0.049)
Renal Necrosis/Cell Death	9.03E-03	22/483 (0.046)
VDR/RXR Activation	5.41E-02	5/78 (0.064)
Increases Renal Proliferation	6.12E-02	7/133 (0.053)

Supplementary Table 8 | Significant terms in Ingenuity Pathway Analysis of age-related genes in humans.

Top Canonical Pathways

Name	*p*-value	Ratio
GPCR-Mediated Integration of Enteroendocrine Signaling Exemplified by an L Cell	9.91E-07	10/71 (0.141)
Factors Promoting Cardiogenesis in Vertebrates	1.08E-05	10/92 (0.109)
GABA Receptor Signaling	4.18E-05	8/67 (0.119)
Wnt/b-catenin Signaling	1.08E-04	12/169 (0.071)
Hepatic Fibrosis / Hepatic Stellate Cell Activation	1.18E-04	13/197 (0.066)

Top Upstream Regulators

Upstream Regulator	*p*-value of overlap
CTNNB1	4.35E-11
POU4F1	8.76E-11
ISL1	3.71E-09
SHH	5.82E-09
OTX2	1.55E-07

Top Diseases and Bio Functions

Diseases and Disorders

Name	*p*-value	# Molecules
Cancer	1.15E-13 - 1.11E-03	359
Gastrointestinal Disease	1.44E-10 - 1.11E-03	278
Developmental Disorder	2.06E-09 - 1.30E-03	84
Psychological Disorders	8.10E-09 - 9.23E-04	50
Organismal Injury and Abnormalities	1.47E-07 - 1.20E-03	190

Molecular and Cellular Functions

Name	*p*-value	# Molecules
Cellular Development	4.65E-08 - 1.43E-03	146
Cell-To-Cell Signaling and Interaction	1.77E-07 - 1.09E-03	50
Molecular Transport	1.90E-06 - 1.21E-03	66
Cell Signaling	4.57E-06 - 1.09E-03	23
Gene Expression	6.04E-06 - 2.04E-04	82

Physiological System Development and Function

Name	*p*-value	# Molecules
Behavior	1.29E-14 - 1.55E-03	78
Embryonic Development	3.08E-13 - 1.43E-03	122
Organismal Development	3.08E-13 - 1.50E-03	136
Nervous System Development and Function	6.65E-13 - 1.55E-03	131

Organ Development	3.40E-10 - 1.43E-03	106

Top Tox Functions

Assays: Clinical Chemistry and Hematology

Name	*p*-value	# Molecules
Increased Levels of LDH	3.28E-02 - 3.28E-02	2
Increased Levels of Albumin	3.80E-02 - 3.80E-02	1
Increased Levels of Creatinine	1.65E-01 - 1.65E-01	2
Increased Levels of Potassium	2.67E-01 - 2.67E-01	1
Increased Levels of Red Blood Cells	5.70E-01 - 5.70E-01	2

Cardiotoxicity

Name	*p*-value	# Molecules
Congenital Heart Anomaly	1.60E-05 - 3.22E-01	15
Heart Failure	2.94E-05 - 1.60E-01	17
Cardiac Stenosis	2.66E-03 - 2.66E-03	5
Cardiac Arteriopathy	2.83E-03 - 2.83E-03	14
Cardiac Congestive Cardiac Failure	4.85E-03 - 4.85E-03	7

Hepatotoxicity

Name	*p*-value	# Molecules
Liver Hyperplasia/Hyperproliferation	8.43E-07 - 4.56E-01	165
Hepatocellular Carcinoma	5.49E-03 - 1.92E-01	23
Liver Cholestasis	1.92E-02 - 5.59E-01	2
Liver Damage	1.92E-02 - 4.44E-01	6
Liver Inflammation/Hepatitis	2.97E-02 - 2.28E-01	9

Nephrotoxicity

Name	*p*-value	# Molecules
Renal Hypoplasia	4.57E-06 - 1.92E-02	8
Renal Dysplasia	1.27E-05 - 7.47E-02	6
Glomerular Injury	4.21E-03 - 3.35E-01	11
Kidney Failure	7.32E-03 - 2.42E-01	10
Renal Atrophy	1.34E-02 - 1.92E-01	4

Top Networks

Associated Network Functions	Score
1 Cell Morphology, Cellular Assembly and Organization, Cellular Function and Maintenance	50
2 Connective Tissue Development and Function, Embryonic Development, Organ Development	47
3 Embryonic Development, Organismal Development, Skeletal and Muscular System Development and Function	36

4 Immunological Disease, Infectious Disease, Neurological Disease	29
5 Cell-To-Cell Signaling and Interaction, Drug Metabolism, Molecular Transport	28

Top Tox Lists

Name	*p*-value	Ratio
Renal Glomerulus Panel (Human)	2.62E-04	4/17 (0.235)
RAR Activation	2.20E-03	10/176 (0.057)
VDR/RXR Activation	3.88E-03	6/78 (0.077)
Increases Cardiac Proliferation	1.16E-02	4/46 (0.087)
Hepatic Cholestasis	1.46E-02	8/165 (0.048)

Supplementary Table 9 | Sites overlapping between age-related methylation in whole blood and cell-type specific methylation.

WB vs Gra (CGI)

	Diff	No Diff	Total
Age	9	1517	1526
No Age	43	11436	11479
Total	52	12953	13005

p=0.21, Chi-square test

WB vs CD34+ cells (CGI)

	Diff	No Diff	Total
Age	158	1608	1766
No Age	191	14095	14286
Total	349	15703	16052

p<0.001, Chi-square test

WB vs T-cells (CGI)

	Diff	No Diff	Total
Age	41	1391	1432
No Age	191	11168	11359
Total	232	12559	12791

p=0.002, Chi-square test

WB vs Gra (nCGI)

	Diff	No Diff	Total
Age	3	3846	3849
No Age	167	9102	9269
Total	170	12948	13118

p<0.001, Chi-square test

WB vs CD34+ cells (nCGI)

	Diff	No Diff	Total
Age	250	4273	4523
No Age	446	10944	11390
Total	696	15217	15913

p<0.001, Chi-square test

WB vs T-cells (nCGI)

	Diff	No Diff	Total
Age	502	3349	3851
No Age	1189	8761	9950
Total	1691	12110	13801

p=0.08, Chi-square test

WB vs Gra (CGI + nCGI)

	Diff	No Diff	Total
Age	12	5363	5375
No Age	210	20538	20748
Total	222	25901	26123

p<0.001, Chi-square test

WB vs CD34+ cells (CGI + nCGI)

	Diff	No Diff	Total
Age	408	5881	6289
No Age	637	25039	25676
Total	1045	30920	31965

p<0.001, Chi-square test

WB vs T-cells (CGI + nCGI)

	Diff	No Diff	Total
Age	543	4840	5383
No Age	1380	19829	21209
Total	1923	24669	26592

p<0.001, Chi-square test

CGI; CpG island
nCGI; non CGI
WB; Whole blood
Gra; Granulocytes
Age; age-related methylated sites in WB
No Age; non age-related methylated sites inWB
Diff; differentially methylated sites between WB and each cell type
No Diff; non differentially methylated sites between WB and each cell type

Supplementary Table 10 | Overlapped genes between the age-related hypermethylated genes identified and those reported in prior publications.

Detectable genes by both DREAM and prior study	Genes hypermethylated by DREAM	Genes hypermethylated in prior study	Genes hypermethylated by only DREAM	Genes hypermethylated in only prior study	Genes hypermethylated by both methods	Prior publications
3228	277 (8.6%)	133 (4.1%)	233	89	44 (33.1% of prior study) (*p*<0.001)	Horvath et al, Genome Biol. 2012
4464	391 (8.8%)	17 (0.4%)	387	13	4 (23.5% of prior study) (*p*=0.03)	Hannum et al, Mol Cell. 2013
3025	256 (8.5%)	46 (1.5%)	245	35	11 (23.9% of prior study) (*p*<0.001)	Horvath, Genome Biol. 2013

p-value; Chi-square test

Supplementary Table 11 | Hypermethylated ARM genes detected by DREAM and in prior studies.

DREAM vs Horvath et al (Genome Biol. 2012)

	Genes hypermethylated in prior study	Genes not hypermethylated in prior study	Total
Genes hypermethylated by DREAM	44	233	277
Genes not hypermethylated by DREAM	89	2862	2951
Total	133	3095	3228

$p<0.001$, Chi-square test

DREAM vs Hannum et al (Mol Cell. 2013)

	Genes hypermethylated in prior study	Genes not hypermethylated in prior study	Total
Genes hypermethylated by DREAM	4	387	391
Genes not hypermethylated by DREAM	13	4060	4073
Total	17	4447	4464

$p=0.03$, Chi-square test

DREAM vs Horvath (Genome Biol. 2013)

	Genes hypermethylated in prior study	Genes not hypermethylated in prior study	Total
Genes hypermethylated by DREAM	11	245	256
Genes not hypermethylated by DREAM	35	2734	2769
Total	46	2979	3025

$p<0.001$, Chi-square test

Supplementary Table 12 | Hypermethylated ARM sites overlapping among species.

Mouse vs Monkey

	Hypermethylated sites in monkey	Non hypermethylated sites in monkey	Total
Hypermethylated sites in mouse	79	29	108
Non hypermethylated sites in mouse	163	513	676
Total	242	542	784

p<0.001, Chi-square test

Mouse vs Human

	Hypermethylated sites in human	Non hypermethylated sites in human	Total
Hypermethylated sites in mouse	50	118	168
Non hypermethylated sites in mouse	68	983	1051
Total	118	1101	1219

p<0.001, Chi-square test

Monkey vs Human

	Hypermethylated sites in human	Non hypermethylated sites in human	Total
Hypermethylated sites in monkey	202	460	662
Non hypermethylated sites in monkey	48	1572	1620
Total	250	2032	2282

p<0.001, Chi-square test

Supplementary Table 13 | Samples used in each analysis and gene selection for pyrosequencing assays and gene expression assays.

		**DNA methylation**											**Gene expression**
		DREAM result (promoter region)			pyrosequencing assay								RT-PCR
					assay established and tested in Bld			common genes among three species for methylation rate in Bld			intra-individual changes in PBMCs	Multiple tissues	TaqMan gene expression assay tested in liver samples
Species		Mouse	Monkey	Human	Mouse	Monkey	Human	Mouse	Monkey	Human	Monkey	Mouse	Mouse
Number of samples	Newborn			5			13			13			
	AL-infant		4			12			12				
	AL-young	5			6		54	6		54		6	6
	AL-middle	7	7	5	13	15	27	13	15	27	3		
	AL-old	7	5	6	12	12	45	12	12	45		12	12
	CR-old	5	6		12	18		12	18		1 (middle)	12	12
Hypermethylated genes (detected by DREAM)													
NPTX2		+	+	+	1	1	1	1	1	1		1	
SOX11		+	+	+	1	1	1	1	1	1		1	
AC009501		+	ND	+	1	1	1	1	1	1			
RIPK4		ND	+	+	1	1	1	1	1	1	1	1	2
ALOX12		ND	+	+	1	1	1	1	1	1		1	
DPYS		ND	+	+	1	1	1	1	1	1			
SORCS1		ND	+	+	1	1	1	1	1	1			
KLF14		-	ND	+	1	1	1	1	1	1		1	
MYOD1		ND	ND	+	1	1	1	1	1	1			
CHSY3		+	ND	-	1	1							
TRHDE		+	ND	ND	1	1						1	
LOC721190		ND	+	ND	1	1							
GATA4		+	+	+		1							
GDNF		+	+	-			1						
WT1		+	ND	+	1							1	
PAX3		ND	+	+	1								
SALL3		ND	+	+		1							
TRIM58		ND	+	+		1							
VWCE		ND	+	+		1							
GATA5		+	ND	-	1								
CAST		ND	+	-		1					1		
ACAP3		ND	+	-		1							
COX6A2		ND	+	ND		1							
DOK2 (LOC706317) *		(ND)	+	(ND)	1								
SOX1		ND	ND	+		1							
TUSC3		ND	ND	+			1						
Hypermethylated genes (detected in prior studies)													
HOXA2		ND	ND	ND	1	1	1	1	1	1			
PGR		ND	ND	ND	1	1						1	
ELOVL2		ND	ND	-	1							1	2
EN2		ND	ND	-	1								
ESR1 *		(ND)	(ND)	(ND)	1								
CDH13		ND	ND	ND	1							1	
GRM7		ND	ND	ND	1							1	
PCDH10		ND	ND	ND	1							1	
DBC1		ND	ND	ND		1							
DLX5		ND	ND	ND		1							
RARB		-	ND	ND			1						
SLC16A12		ND	ND	-			1						
Hypomethylated genes (detected by DREAM)													
MSLN		ND	+	+	1	1	1	1	1	1			
RILP		+	ND	-	1	1	1	1	1	1			

EIF4EBP3		+	ND	ND	1	1							
LOC703422		ND	+	ND	1	1							
FNBP1 **		(ND)	+	(ND)	1	1					1		
LIMS2		ND	+	-	1							1	2
TAPBP		+	ND	ND	1							1	2
CKM		+	ND	ND	1							1	
4933407P14RIK		+	ND	ND	1								
GM5414		+	ND	ND	1								
GOLT1A (LOC700305)		ND	+	ND		1							
LOC695235		ND	+	ND		1							
LOC701241		ND	+	ND		1							
NCS1 (LOC722507)		ND	+	ND		1							
VSIG2 (LOC712047)		ND	+	ND		1							
DOK2 (LOC706317) *		(ND)	+	(ND)		1							
SORBS2 ***		(ND)	+	(ND)		1							

Bld; whole blood
PBMC; peripheral blood mononuclear cell
AL; ad libitum
CR; caloric restricted
+; age-related methylation
-; non age-related methylation
ND; not detectable
1; pyrosequencing assay performed
2; TaqMan assay performed
*; 2nd exon
**; 3' end region
***; gene body

Supplementary Table 14 | Summary of bisulfite pyrosequencing assays in mouse whole blood.

Gene	Spearman *r*-value	*p*-value (2-tailed)	Mean of methylation % (±SEM) Y	M	O	CR	O - Y	CR - O	t-test (Y vs O)	t-test (O vs CR)
Hypermethylated genes										
Ac009501-py1	0.78	<0.001	6.6±0.4	10.6±1.6	11.8±0.7	9.3±0.8	5.2	-2.6	<0.001	0.03
Elovl2-py1	0.71	<0.001	12.4±1.6	20.4±1.8	25.0±2.0	22.4±2.2	12.5	-2.6	<0.001	0.38
Trhde-py2/py3/py4	0.65	0.003	13.3±0.4	21.0±1.3	36.4±4.3	21.9±2.1	23.1	-14.5	<0.001	0.008
Grm7-py1	0.63	0.005	21.5±1.4	34.0±1.6	51.4±5.9	31.5±2.1	29.9	-19.9	<0.001	0.007
Sox11-py1/py3	0.62	0.006	16.0±1.5	24.9±2.0	41.1±5.2	19.2±1.8	25.1	-21.9	<0.001	0.002
Dok2-py1/py2	0.58	0.01	20.5±1.2	34.5±3.0	44.3±5.1	28.7±2.8	23.7	-15.6	<0.001	0.02
Klf14-py1/py2	0.56	0.02	18.9±1.0	29.8±0.8	31.5±2.9	29.6±1.3	12.6	-1.9	0.001	0.56
Pcdh10-py1	0.55	0.02	12.7±0.9	19.1±1.6	34.2±5.2	14.5±1.2	21.5	-19.8	0.002	0.003
Loc721190-py1	0.54	0.02	11.3±1.4	15.8±1.1	19.8±2.3	13.3±1.3	8.4	-6.5	0.007	0.02
Nptx2-py2/py4	0.54	0.02	10.3±0.2	12.1±0.5	21.1±2.8	13.0±0.9	10.8	-8.1	0.003	0.02
Wt1-py1	0.52	0.03	10.1±0.6	13.4±0.8	18.2±2.2	13.2±1.1	8.2	-5.1	0.004	0.05
Dpys-py1/py2	0.52	0.03	19.9±0.7	21.1±1.0	31.8±3.0	20.6±1.1	11.9	-11.2	0.003	0.004
Ripk4-py2	0.50	0.04	9.1±1.3	11.8±0.8	14.0±1.5	8.8±0.7	4.9	-5.3	0.03	0.007
Pgr-py4	0.49	0.04	10.7±0.5	14.1±1.0	22.7±3.6	13.0±0.7	12.0	-9.7	0.007	0.02
Cdh13-py1	0.48	0.04	10.9±0.4	14.1±1.1	20.9±3.1	13.1±1.0	10.0	-7.7	0.008	0.03
Chsy3-py2	0.48	0.04	6.5±0.8	12.2±1.9	28.3±6.6	11.6±1.1	21.8	-16.7	0.007	0.03
En2-py1	0.42	0.08	5.5±0.6	8.2±0.7	9.1±1.2	7.4±1.3	3.5	-1.7	0.02	0.35
Sorcs1-py2	0.40	0.09	7.7±2.8	13.8±1.5	27.3±7.4	12.3±2.7	19.6	-15.0	0.03	0.08
Alox12-py1	0.40	0.10	6.2±0.2	8.0±0.2	9.9±1.4	7.1±0.2	3.7	-2.8	0.02	0.08
Myod1-py5/py6	0.37	0.13	7.6±0.5	10.4±0.4	10.3±1.1	7.9±0.5	2.7	-2.4	0.04	0.08
Gata5-py1	0.32	0.19	11.2±0.7	11.4±0.9	14.0±1.5	12.5±1.2	2.8	-1.4	0.11	0.45
Pax3-py1	0.28	0.26	6.3±1.0	5.9±0.9	9.8±1.8	6.6±1.2	3.5	-3.2	0.11	0.16
Esr1-py4	0.27	0.27	60.2±2.1	63.6±2.1	65.6±3.7	51.0±2.7	5.5	-14.6	0.22	0.004
Hoxa2-py1	0.22	0.38	28.8±1.5	29.1±1.1	31.1±1.5	25.8±1.3	2.3	-5.3	0.28	0.01
Ave of 24 genes	0.64	0.004	14.3±0.3	19.1±0.7	26.2±2.3	17.3±0.7	11.9	-9.0	<0.001	0.003
Ave of common 10 genes	0.61	0.008	13.1±0.4	17.2±0.5	23.0±2.1	15.4±0.6	9.9	-7.6	<0.001	0.005
Hypomethylated genes										
Rilp-py1	-0.58	0.01	89.7±0.9	72.5±3.2	69.8±4.3	74.0±4.1	-19.9	4.2	<0.001	0.49
Gm5414-py1	-0.56	0.02	86.2±1.2	79.6±0.9	57.6±6.7	61.7±5.2	-28.6	4.1	0.002	0.64
Fnbp1-py2	-0.52	0.02	97.3±0.2	93.7±0.2	94.1±0.8	95.5±0.5	-3.2	1.4	0.004	0.15
Tapbp-py2/py3	-0.51	0.03	81.6±0.4	71.7±2.2	60.9±5.6	72.9±1.6	-20.8	12.0	0.003	0.05
Ckm-py1/py2/py3	-0.45	0.06	73.9±1.3	63.7±2.9	50.2±7.1	70.6±4.1	-23.6	20.4	0.007	0.02
Eif4ebp3-py1	-0.45	0.06	89.6±2.3	75.8±3.0	61.6±8.1	79.7±3.5	-28.0	18.1	0.006	0.05
Lims2-py1	-0.39	0.11	40.6±1.0	37.0±1.4	31.5±3.6	37.2±2.3	-9.2	5.7	0.03	0.20
Loc703422-py4	-0.38	0.12	88.4±0.9	85.0±1.4	79.9±3.3	84.1±1.2	-8.5	4.2	0.03	0.26
4933407P14Rik-py2	-0.38	0.12	51.2±1.0	46.0±0.9	39.1±4.5	44.5±2.2	-12.1	5.4	0.02	0.30
Msln-py1	-0.28	0.27	92.6±1.0	88.1±2.0	88.8±2.0	90.6±0.9	-3.8	1.8	0.11	0.43
Ave of 10 genes	-0.51	0.03	79.1±0.4	71.3±1.2	63.3±4.1	71.1±1.1	-15.8	7.7	0.003	0.09
Ave of common 2 genes	-0.52	0.03	91.2±0.6	80.3±2.6	79.3±3.0	82.3±2.2	-11.9	3.0	0.003	0.43

Y; AL-young
M; AL-middle
O; AL-old
CR; CR-old
t-test; Unpaired t test with Welch's correction

Supplementary Table 15 | Summary of bisulfite pyrosequencing assays in monkey whole blood.

Gene	Spearman r-value	*p*-value (2-tailed)	Mean of methylation % (±SEM) I	M	O	CR	O - I	CR - O	t-test (M vs O)	t-test (O vs CR)
Hypermethylated genes										
SALL3-py1	0.87	<0.001	6.6±0.3	14.5±1.5	21.9±1.5	20.5±1.7	15.3	-1.4	0.002	0.54
PGR-py2	0.86	<0.001	5.4±0.5	13.2±1.3	20.0±1.2	17.7±1.6	14.6	-2.3	<0.001	0.27
NPTX2-py1	0.85	<0.001	15.0±0.8	24.5±1.3	33.0±1.4	30.2±2.1	18.0	-2.8	<0.001	0.29
SOX1-py2/py3/py4	0.85	<0.001	12.0±0.8	25.4±1.9	33.3±1.5	30.5±1.9	21.4	-2.9	0.003	0.24
GATA4-py1	0.82	<0.001	8.9±0.2	13.1±0.8	15.9±0.7	16.0±1.2	7.1	0.1	0.02	0.97
SORCS1-py7	0.82	<0.001	15.0±0.7	34.6±2.9	44.8±2.4	42.2±2.5	29.8	-2.7	0.01	0.45
AC009501-py2	0.80	<0.001	13.4±0.5	19.4±1.5	24.4±0.8	21.2±1.1	10.9	-3.2	0.008	0.03
KLF14-py1	0.79	<0.001	9.9±0.5	12.6±0.6	15.4±0.9	14.0±0.9	5.6	-1.4	0.01	0.27
DBC1-py2	0.78	<0.001	15.5±0.7	23.2±1.8	28.1±1.0	27.5±1.3	12.5	-0.6	0.03	0.74
VWCE-py2	0.78	<0.001	31.6±0.8	38.3±1.7	44.9±1.6	43.0±1.7	13.2	-1.9	0.01	0.43
MYOD1-py2	0.77	<0.001	13.6±0.6	22.6±1.4	27.4±1.3	24.1±1.3	13.8	-3.3	0.02	0.09
SOX11-py2	0.77	<0.001	10.7±1.0	22.4±2.2	28.4±1.4	26.2±2.3	17.7	-2.2	0.03	0.40
CAST-py1	0.75	<0.001	19.7±3.0	57.6±2.4	65.7±1.7	58.1±2.9	46.0	-7.6	0.01	0.03
TRIM58-py3	0.74	<0.001	4.8±0.3	7.7±1.1	12.3±1.0	10.6±0.8	7.5	-1.7	0.005	0.20
RIPK4-py1	0.73	<0.001	8.1±0.5	9.7±0.7	12.7±0.6	11.1±0.5	4.6	-1.6	0.003	0.06
TRHDE-py2/py5	0.69	<0.001	7.5±0.5	8.6±0.9	13.2±0.6	10.6±0.9	5.7	-2.6	<0.001	0.02
DLX5-py1/py2	0.67	<0.001	9.8±0.4	10.9±0.7	14.2±0.8	13.2±0.7	4.4	-0.9	0.006	0.41
CHSY3-py1	0.64	<0.001	4.6±0.3	4.7±0.6	8.9±1.0	7.8±0.8	4.3	-1.1	0.002	0.40
ALOX12-py1	0.60	<0.001	11.7±0.6	11.7±0.9	17.1±1.5	15.7±1.0	5.4	-1.3	0.006	0.47
HOXA2-py1	0.53	<0.001	14.4±0.6	17.7±0.6	17.9±1.0	16.8±0.9	3.5	-1.1	0.86	0.43
ACAP3-py2	0.48	0.002	23.9±1.2	26.5±1.8	32.1±2.0	25.5±1.8	8.2	-6.6	0.04	0.02
COX6A2-py1	0.45	0.004	44.1±3.0	47.0±2.3	55.7±3.1	46.6±3.3	11.7	-9.1	0.03	0.06
LOC721190-py2	0.38	0.02	26.0±1.6	29.1±1.8	35.5±3.1	31.2±2.3	9.5	-4.3	0.09	0.28
DPYS-py1/py4	0.12	0.46	21.2±1.2	21.1±1.4	22.7±1.7	22.6±1.7	1.5	-0.1	0.47	0.37
Ave of 24 genes	0.87	<0.001	14.7±0.5	21.5±1.0	26.9±0.7	24.3±0.9	12.2	-2.6	<0.001	0.04
Ave of common 10 genes	0.85	<0.001	13.3±0.5	19.6±1.1	24.4±0.8	22.4±0.9	11.1	-2.0	0.002	0.10
Hypomethylated genes										
VSIG2-py1/py2	-0.81	<0.001	82.5±0.9	71.0±1.4	66.6±1.6	68.5±1.7	-16.0	1.9	0.05	0.42
LOC701241-py3	-0.80	<0.001	84.0±0.7	70.0±1.8	66.4±1.4	68.6±2.4	-17.6	2.2	0.13	0.43
LOC695235-py1	-0.79	<0.001	73.3±0.7	60.3±1.4	58.0±1.8	59.0±1.4	-15.3	1.0	0.32	0.67
GOLT1A-py1	-0.78	<0.001	88.5±0.7	70.2±2.3	66.8±2.0	67.7±2.6	-21.7	0.9	0.27	0.77
DOK2-py1	-0.76	<0.001	72.1±1.5	50.0±2.5	44.5±2.4	48.9±2.0	-27.6	4.4	0.13	0.17
MSLN-py2/py3	-0.75	<0.001	80.4±1.2	63.6±2.1	62.3±1.5	64.0±1.6	-18.1	1.7	0.60	0.45
FNBP1-py2	-0.75	<0.001	91.3±0.6	80.6±2.1	73.4±2.6	79.1±1.7	-17.9	5.7	0.04	0.08
LOC703422-py1/py2	-0.55	<0.001	81.9±0.5	80.1±0.4	77.1±1.6	79.5±0.5	-4.8	2.5	0.10	0.17
RILP-py1	-0.43	0.007	91.7±0.3	88.1±1.8	89.2±0.7	90.0±0.3	-2.4	0.7	0.58	0.34
EIF4EBP3-py3	-0.32	0.04	86.8±0.5	85.3±0.4	85.1±0.8	85.1±0.3	-1.8	0.0	0.81	0.98
NCS1-py2	-0.26	0.09	94.3±0.6	89.1±2.5	91.2±1.2	91.5±0.8	-3.1	0.3	0.46	0.84
SORBS2-py1	-0.25	0.12	25.7±1.3	25.2±1.3	22.7±1.9	23.2±1.7	-3.0	0.5	0.29	0.85
Ave of 12 genes	-0.81	<0.001	79.4±0.4	69.5±1.1	66.9±1.1	68.8±1.1	-12.4	1.8	0.13	0.25
Ave of common 2 genes	-0.71	<0.001	86.0±0.6	75.9±1.3	75.7±0.8	77.0±0.8	-10.3	1.2	0.93	0.28

I; AL-infant
M; AL-middle
O; AL-old
CR; CR-old
t-test; Unpaired t test with Welch's correction

Supplementary Table 16 | Summary of bisulfite pyrosequencing assays in human whole blood.

Gene	Spearman *r*-value	*p*-value (2-tailed)	Mean of methylation % (±SEM) N	Y	M	O	O - N	t-test (N vs O)
Hypermethylated genes								
DPYS 4CG	0.60	<0.001	7.3±0.2	13.0±0.3	15.5±0.5	15.9±0.5	8.6	<0.001
NPTX2-py3	0.58	<0.001	16.0±0.9	25.1±0.6	28.5±1.0	30.6±0.9	14.5	<0.001
SLC16A12 4CG	0.58	<0.001	3.7±0.6	6.5±0.2	13.2±2.6	11.0±0.8	7.3	<0.001
SOX11-py1	0.45	<0.001	5.2±0.4	9.6±0.7	11.7±0.9	12.4±1.0	7.2	<0.001
TUSC3 4CG	0.42	<0.001	2.9±0.2	11.3±0.6	14.5±1.3	16.1±1.7	13.1	<0.001
ALOX12-py5	0.33	<0.001	26.5±2.5	34.9±1.5	45.3±2.0	41.1±2.1	14.6	<0.001
HOXA2-py3	0.33	<0.001	27.3±2.0	41.3±1.4	41.8±2.4	43.6±1.3	16.3	<0.001
RARB	0.33	<0.001	3.2±0.2	3.6±0.1	4.7±0.4	4.4±0.3	1.2	0.002
MYOD1	0.31	<0.001	6.9±0.2	13.2±0.5	16.6±1.3	14.8±0.8	7.9	<0.001
AC009501-py6	0.29	<0.001	9.3±0.6	18.4±1.6	16.9±1.6	21.3±2.0	12.0	<0.001
RIPK4-py1	0.27	0.003	6.4±0.5	10.4±0.4	12.9±1.2	12.3±0.9	5.9	<0.001
GDNF	0.24	0.006	3.4±0.1	6.5±0.3	8.2±0.8	7.1±0.6	3.7	<0.001
KLF14-py2	0.18	0.07	32.8±1.9	40.4±0.8	44.4±1.6	42.2±1.6	9.4	0.001
SORCS1-py4	0.07	0.40	20.2±2.2	27.7±0.9	26.5±0.7	26.2±0.5	6.0	0.02
Ave of 14 genes	0.56	<0.001	12.1±0.5	18.8±0.3	21.5±0.6	21.6±0.5	9.5	<0.001
Ave of common 10 genes	0.51	<0.001	16.1±0.8	24.0±0.5	26.5±0.6	26.9±0.7	10.7	<0.001
Hypomethylated genes								
RILP-py1	-0.44	<0.001	87.5±1.4	73.9±0.9	71.0±1.5	70.1±1.4	-17.4	<0.001
MSLN-py1	-0.39	<0.001	95.5±0.5	88.0±1.2	84.7±1.1	87.6±0.9	-7.8	<0.001
Ave of 2 genes	-0.43	<0.001	91.1±0.9	79.0±1.0	72.8±1.8	74.7±1.6	-16.3	<0.001

N; Newborn
Y; Young
M; Middle
O; Old
t-test; Unpaired t test with Welch's correction

Supplementary Table 17 | Summary of age-related methylation analyses using pyrosequencing assays in mixed effect model.

**Multilevel mixed linear model**					
Species	Tissue	Slope (±SE)	*p*-value	Intercept (±SE)	*p*-value
**Common 10 hypermethylated genes**					
Mouse	Whole blood	4.12±1.16	<0.001	11.89±3.50	<0.001
Monkey	Whole blood	0.34±0.14	<0.001	13.60±3.67	<0.001
Human	Whole blood	0.10±0.02	<0.001	20.06±3.13	<0.001
**Common 2 hypomethylated genes**					
Mouse	Whole blood	-4.20±1.06	0.004	89.89±2.15	<0.001
Monkey	Whole blood	-0.25±0.09	<0.001	83.74±12.70	<0.001
Human	Whole blood	-0.12±0.02	<0.001	85.77±5.20	<0.001
**All 24 hypermethylated genes**					
Mouse	Whole blood	5.09±0.38	<0.001	12.82±2.64	<0.001
Monkey	Whole blood	0.47±0.02	<0.001	15.05±2.40	<0.001
**All 14 hypermethylated genes**					
Human	Whole blood	0.09±0.01	<0.001	15.67±3.44	<0.001
**Common 12 hypermethylated genes**					
Mouse	Whole blood	6.62±0.78	<0.001	11.79±3.58	0.001
Mouse	Spleen	5.22±0.51	<0.001	12.35±3.21	<0.001
Mouse	Bone marrow	2.74±0.23	<0.001	11.29±2.59	<0.001
Mouse	Liver	3.13±0.47	<0.001	8.17±1.88	<0.001
Mouse	Kidney	1.40±0.27	<0.001	5.84±0.92	<0.001
Mouse	Small intestine	6.04±0.54	<0.001	8.63±3.97	0.03
Mouse	Large intestine	9.52±0.57	<0.001	8.31±4.43	0.06

Slope; Methylation rate (%/year)
Intercept; Methylation (%)
SE; Standard error
Formulas for age prediction;
Age(year) = (1/Slope) x Methylation(%) + (Intercept/Slope)

Supplementary Table 18 | List of genes for which the methylation rate by age is significantly affected by CR.

**Mouse**		**Monkey**	
Negative coefficient (decrease methylation drift) 111 genes	Positive coefficient (increase methylation drift) 26 genes	Negative coefficient (decrease methylation drift) 103 genes	Positive coefficient (increase methylation drift) 13 genes
5930412G12Rik	4933403G14Rik	ACAP3	ARHGAP27 ****
Aard	4933407P14Rik	ADAMTS16	LOC100426511
Adcy1	Adamtsl4	ADCY2	LOC100427157
Agap1	Apba3	ADCY5	LOC100430847 ****
Ano5	Bcl7b ****	ADCYAP1R1	LOC701241
Arhgef5	Ccdc74a	CA4	LOC703422 ***
Astn2	Ckm	CAST	LOC706559
Atp11a	Ctnna3	CCNA1	LOC712918 **
Atp9a	Derl3	CDH11	LOC716530 ****
Bcl9l	Dtx1	COL2A1	LOC722432
Bnc2	Eif4ebp3	COL4A3	LOC722507
Car10	Espnl	CRHR2	SOHLH2
Cbln2	Fes	CTSL2	TGM1
Cdh4	Ficd	CTTNBP2	
Chst2	Iqce	CXCL3	
Chsy3	Muc20	DACT2	
Cnih3	Pgf	DAPK1	
Cnr1	Rasgrp4	DNMT3A	
Colec12	Rhod	EPHA5	
Ctnnd2	Rilp	FAT1	
Cttnbp2	Smcr7	FLRT2	
Cxadr	Spg21	FREM2	
Dab1	Stk33	FUT4	
Dazl	Tapbp	GABRA2	
Dock5	Tmem171	GABRA5	
Epb4.1l3	Ttbk1	GALNT14	
Ephb3		GDNF	
Fam174b		GOLSYN	
Fam181b		GPC5	
Fam189a1		GRB10	
Fam189a2		HHEX	
Fam38b		IGDCC3	
Fbxl7		IRX1	
Fndc3b		IRX3	
Frmd5		IRX5	
Fscn1		KDR	
Fzd10		KLF4	
Galnt13		LOC100424606	
Gata4		LOC100425129	

Gdpd3 *	LOC100429790
Gpr125	LOC100430337
Gpr126	LOC100430564
Grb10	LOC693425
Gria4	LOC695843
Hoxd1	LOC697860
Hs6st3	LOC698887
Htr1b	LOC700998
Hunk	LOC701087
Igsf11	LOC702481
Irx3	LOC705490
Ism1	LOC705699
Kcnd3	LOC705851
Kcnma1	LOC707576
Klhl29	LOC708182
L3mbtl4	LOC709762
Lhfpl2	LOC711708
Lrig1	LOC713545
Lrig3	LOC716399
Lrp5	LOC718232
Lypd3	LOC718355
Mal2	LOC722961
Med12l	LRRK2
Mme	LTBP1
Mpped2	MAN1C1
Myrip	MPPED2
Nkain2	MTMR7
Nox4	MYO10
Npas2	MYO3A
Npnt	NETO1
Nr2f2	NEUROG1
Ntsr2	PAX3
Otx1	PCDH9
Pam	PPP1R9A
Pclo	PTH2R
Phyhipl	QPCT
Plcb1	RGS22
Ppargc1b	RGS7
Ppfia2	RNF180
Ppp1r3d	RSPO2
Prdm5	SCIN
Ptprg	SH2D4A
Rgs3	SH3GL3
Rgs6	SLC16A14
Rhoj	SLC1A1
Rnf217	SMTNL2

Runx1t1	SNX22
Sh3bp4	SORCS1
Shc3	SPAG6
Slc7a10	ST3GAL6
Slc9a2	ST6GALNAC2
Slitrk5	ST8SIA5
Smo	STARD13
Snap91	SYDE2
Sorcs3	TJP1
Sox11	TLX3
Sox9	TMEM37
Stc2	TRIM58
Tcfl5	TRPM2
Tlx3	UACA
Tmem229a	WNT11
Tmem90b	ZFHX3
Trhde	ZNF141
Tspyl5	ZNF667
Twist2	
Ugt8a	
Vangl1	
Vldlr	
Xkr7	
Zdbf2	
Zfp385b	
Zfpm2	

Coefficient calculated by the linear model shows the effect of CR on aging methylation drift
t-test for coefficient; p<0.05
promoter (-1kb<TSS<+500bp)
average of methylation≥1%
Negative (positive) coefficient means that CR tends to decrease (increase) aging methylation drift.
All genes, except 7 genes, with negative (positive) coefficient showed age-related hypermethylation (hypomethylation).
*; gene with negative coefficient showed age-related hypomethylation.
**; gene with positive coefficient showed age-related hypermethylation.
***; gene with positive coefficient showed both age-related hypermethylation and hypomethylation.
****; non age-related gene
Binomial pvalue; p<0.001 (in each species)

Supplementary Table 19 | Comparison of age-related methylation in mouse multiple tissues.

Methylation differences (AL-old minus AL-young)

	Bld	Spl	BM	Lvr	Kid	SI	LI
Hypermethylated genes							
Trhde-py4	35.5	28.9	13.0	7.6	3.7	39.2	50.0
Grm7-py1	29.9	23.4	10.5	17.1	9.3	39.5	43.9
Sox11-py1	22.6	16.4	4.4	5.2	3.6	4.2	5.2
Pcdh10-py1	21.5	15.1	3.9	4.9	2.0	17.9	36.3
Klf14-py1	14.0	13.7	11.1	10.6	9.1	13.2	25.1
Elovl2-py1	12.5	8.5	14.4	5.6	4.1	7.3	25.3
Nptx2-py4	12.2	6.5	3.1	7.3	2.0	3.5	8.2
Pgr-py4	12.0	8.3	3.0	4.0	2.0	1.0	20.4
Cdh13-py1	10.0	13.5	5.2	3.4	2.3	7.1	14.0
Wt1-py1	8.2	6.2	4.3	6.8	2.8	26.2	26.5
Ripk4-py2	4.9	4.1	1.8	5.1	0.6	1.3	2.1
Alox12-py1	3.7	3.1	1.0	1.9	0.8	1.9	2.2
Ave of 12 genes	15.6	12.3	6.5	6.6	3.6	13.6	21.6
Hypomethylated genes							
Tapbp-py3	-26.4	-26.0	-0.7	-1.4	-20.7	-5.4	-2.3
Ckm-py1	-22.5	-16.7	-6.9	-5.2	-4.6	-7.1	-2.6
Lims2-py1	-9.2	-11.9	0.0	-4.1	-13.1	-5.2	-9.1
Ave of 3 genes	-19.4	-18.2	-2.5	-3.6	-11.9	-5.9	-4.5

p<0.05

p<0.01

Unpaired t-test with Welch's correction

Bld; Blood
Spl; Spleen
BM; Bone marrow
Lvr; Liver
kid; Kidney
SI; Small intestine
LI; Large intestine

Supplementary Table 20 | Primer sequences and PCR conditions for bisulfite pyrosequencing analysis.

	1st PCR				Pyrosequencing		
Gene	Forward primer	Reverse primer	Size (bp)	Annealing	Sequence primer	Analyzed sites	Sequence region
Mouse							
Ac009501-py1	GAGGGATTTTAGGTATTGTGTTGA *	AAACCCCTCCTACTTAATTCCC	146	60	ATTGTGTTGAGTTGTAGTAA	3	YGYGATTTTTAGGTTGYGGTTTAAAT
Alox12-py1	TTGTGGTTGGTTGGGGAGTAT	TCCTATCCCTTACCATCCTCCTAC	133	60	GGAGAAATTGGAGTTGTAA	3	TTGYGGTTAGYGYGGGGTAAG
Chsy3-py2	TAGGGGGGAGATGAGTTAGGA *	ACACCCCCCACTCTACCTCTATAT	128	60	ATGTTAAGTTTATTTATGGG	5	YGGYGGGAGYGTAYGGTTATYGTAG
Dok2-py1	GTGGTATATTTGGGTTTAGAAGTG	AACCTTCCCAATAACCTCTCCTC	133	65	TATATTTGGGTTTAGAAGTG	4	GYGTYGGTTYGTAGTYGTGTTA
Dok2-py2	TGTTATATGGAGAGTTTGGTTGTG	CCCTCTACCCAAAAAACTACCAAT	220	60	AGGATGTTTTAGAGAAGATA	2	YGGYGAGGAGAGGT
Dpys-py1	GGGAGAGGGAGTTTTGGTATAGTT *	CAAATACCTCCTCAAAAAACAACA	218	60	TGGATTATGGTATTATAGGG	4	AYGATTTTTTATTYGYGGGGGTYGTATTGTTAATGA
Dpys-py2	GTTTTTGTGGTGTTATGGTTTAGG *	CCTCACCTACCACCAAAAATCT	135	60	GGTGTTATGGTTTAGGG	2	YGTTGGGGATTGYGAATAAAGTTGGT
Klf14-py2	GGAGAAGTGTTAGAGGGGTTTTTA *	ATCCCAATCCAACCAATCACA	210	60	GAAGTATAGAAGGAGGTAGA	3	TTAYGTTYGTATTTAAGYGATA
Loc721190-py1	TTTTGGAGTGTTGAGGAGGAGTA	CAACTAACACCCAAAACCAAAAAT	264	60	GAAGAGGTAGTTGGTAGAAA	3	GYGYGAGYGGTTTATA
Myod1-py5	GAGAGTAGTAGGGGTTTGTTAAGTTTATGT	CAACCCACAAAACCTTTATCATTATAC	161	65	GGGTTTGTTAAGTTTATGTT	3	TYGGAGTGGYGGYGATAGAAGTT
Myod1-py6	AGAAGATTTTTAAGGTTTGGGTTGAG	AATCCAAACACCAATCATCATAAA	156	55	TTGGGATATGGAGTTTT	3	TATYGTYGTTATTTYGGGATA
Nptx2-py2	AAGGAGTTTTGGGGATTTTGTA *	CCCCCCCTTTCTTAAACTTAA	192	60	GTAGGAGGATTAATTAGAGA	6	GYGYGGYGYGAGTTTTAYGYGTTTGT
Nptx2-py4	GGGTTTGGGATTTTGGGTA	TCCAACCCCAATCTTCCA	126	60	TAGAGTTAGAATAAGGAAGT	6	TTAYGYGAGTYGYGTAGGTGGTTYGYGTGGAAGATT
Ripk4-py2	GAGGGAGGGGTATGTATTTGAGTA	ACCCACCTTCTCCCAACCTAC	156	60	AGGTTGAGTTAGGTTGTTT	4	GYGATAYGTAYGYGATGGA
Sorcs1-py2	GGGGTGGAGTAGAATTGAAGTTA *	CCCCTACTCCCAAAACCTAAAACT	248	60	AGTTAAGGGAGGAGAGG	5	GYGTYGYGYGTYGGTTGG
Sox11-py3	GAGGGGAGGAGATGTTGAGTTT *	CTCATCCTCCCATTCTTTTTCTC	263	60	TGAGTTTAGGGTTGTTTTAT	5	YGATTYGGTTTGAATTYGTGAATTTTTTTTYGAATYGTTT
Trhde-py2	AGTAGGTTGGGATTTTAGGGAG	AACCACACCCTTCCCAAATT	167	60	AGATTTTGGAGGTTAGTGA	3	YGATYGAGAYGATTTTTATTAGTTT
Trhde-py3	GGGGGTTGGTGAAGTTTTAATTT	CTAAAACCCCCCCCAACTTAA	246	60	GAGTAGTAGGAGTATAGGG	6	GYGGGGGYGGYGTGTGTTYGGGYGTTYGGTGTTGGTT
Trhde-py4	AGAGAGGAGGGGTTGATGGG	CCCCTCCAAAACCATCACAC	222	60	GAGTGAGGTTTTTAATGATT	2	GYGTTGGAGGTGGTTYGGGTGGTT
4933407P14Rik-py2	GAAGTGGGGTATGGTGTAGGTTT	CCTCCACCCCTTAAACTCACT	176	60	TTTGTTTTAGTTTTTTGATA	4	YGATTYGGATTAGGTYGTATTTTTTTYGGGTTT
Ckm-py1	TAAGGTTATGGGGTTGGGTAAGTT *	CCCCAAAAAAAAACCCCTAAAAAT	104	60	GGGTTGGGTAAGTTGTA	4	YGTTTGGGTTYGGGGTGGGTAYGGTGTTYGGGTAA
Ckm-py2	TTTGGGGATAGTTTTTTTTGGTT *	TCCCACCCCAATAATTTCTCT	160	60	GGGTATAGGGGTTGTTT	1	TYGGGTTATTATTATTTTTATAGTATAGATAGA
Ckm-py3	TTTAGGGAGGGGTTTTTGAGA *	CCCTTAAAACAACCCATACAAAA	121	60	TGAGAGTAGATGAGTTTTTA	3	GTTYGTTGTTYGGGTATYGTGTTTATTT
Eif4ebp3-py1	TGGAAAATAGTTTTGAGGTGTAGG	TTACTCCTCCCAACATCCAAATCT	203	60	AATAATTAGAATATGTTATA	1	TTYGGGGTAGGTTATTGGTTAGT
Gm5414-py1	TGGTTTTAGTTGTGTGTTTTTGTG *	CCAAAATCTCCCCCATAATATCT	183	60	TGTGTGTTTTTGTGTTAATT	1	TYGGGGTATTAGTGGTTTTGGGT
Loc703422-py4	GGGGAGGGTAAAATTGATGTTAA	ATCCTCCAACCATCTAACTTCTCC	178	60	TGTTTTTTTGGGTAGTAGTG	2	YGTTTTATGGTYGGTTTATTTTTTTTT
Msln-py1	TAGTGGGGTATTTTTGTTGTGAGG	ATTCCCAACCTAAACCACTCAATA	170	60	ATTTTTTGTTTAGGTTGTTA	3	YGGGTTYGTYGTTTGTATTA
Rilp-py1	GTTGAGGATTAGAGTTGTAAGTGG	CCTTCAATCTTTCACCTCTTAACA	106	60	TAGGTAAGTTTTTTTTATAG	2	YGTTTGATTYGGGTTTTATTTTTGGATTTG
Tapbp-py2	TTTATGTGGAGGGATAGTTTTATG	CCCCCACCCACTATAAAAAACTC	164	60	GTTATAGGGGATATTGTGAG	3	YGTYGGTATYGGATGGGTGTGGGTGGGGT
Tapbp-py3	TGTGTAAGTTGGGTGGGGATTAG *	CCAAACACCTTCTAAACCAACATT	131	60	TGGGTGGGGATTAGAT	4	AYGTTTATGGGAYGGGGGYGTGGTYGAATTTGTTTTAT
Fnbp1-py2	TGTTGGATGATAGGGAAGGTTTGA	ACCCAAACCACCAAAACAACT	286	65	TTGGATATGAAGGATATATT	2	TGAYGATGAGTTTTAAGAGAYGTAT
Monkey							
M-AC009501-py2	GAGAGTATTAGTTTTGGGGAAAAG *	AAAAAACACCCACCTCTACACTAA	147	60	TTTTGGGGAAAAGAATA	4	TGYGGAYGTTTYGGGGTGYGTAT
M-ACAP3-py2	TGGTTTTAGTTTAGGTTTTGTGG *	AACACCTAACCACCCCCTCC	146	60	GGTTTTGTGGTGTTTAATTT	3	TYGGGAGGTTAGATGGTTYGGTYGGTTAG
M-ALOX12-py1	TTAGTTTTAATTTAGGGGTTTTTG *	AATACCACTAACTTTCCTAAACCT	72	60	AGGGGTTTTTGTTTATTT	3	YGATTTYGTTTTTYGGGAGG
M-CAST-py1	GGTAGGAAGTTGGGGGATAT	CAAACCCAAATTTCCAAAATCT	116	60	AAGTTGGGGGATATAAAT	6	YGTTYGATTYGGGAGTAYGYGYGGTTTGG
M-CHSY3-py1	GGATGGGAAGGGAAATGG	CCTCACCCCCCCTTAAAC	163	60	GGTAGGTTTTGGATTGAG	4	GYGYGGAYGGGGGTYGTTTAAGGG
M-COX6A2-py1	ATTGGTTTAGAGAGGGGAGGG *	CCCCATTCACCTACTAACCCTTC	164	65	TGTTTTTGAGGTTTTTGAT	3	TYGGGGTTTGGTTAGYGTTGTTAAAGGAGGTTAYGAAGGGTT
M-DBC1-py2	GGAGGAGGAGAAGTTAGAGGATGA	CATACCCACCATCCCCTTAAAT	155	60	AGAAGTTAGAGGATGAGAGA	5	YGGGAGGTYGTTTYGAGGGAAGGGYGGTTYGGGTGGG
M-DLX5-py1	GGTTTAGTAGGGAGTTATTTAGTTAAAGTTTA	CTCTCTTTATCCAAAAATCTAAATCCTACTC	264	55	GATTATAGTTAAGTTAGTTTTTATTATTAG	5	TAYGGYGGYGTTTATAATYGTATTTTAAGYGTTA
M-DLX5-py2	GATAGGTTGGGTAGATATA	TACCCAATACCCCCCCAC	102	60	GATATAGGGGGATTAGG	3	YGTTGGGGGYGYGAGA

	GGGGGATTA	TACA			A		
M-DPYS-py1	TGGGGGTGTTTTTTTGTAAAGTT *	AAATCTTTCCCTTCCAATAACTCC	254	60	TTATGATTTGGTAGTATATG	2	YGGTTTTTTTTTYGGGAAGGTTG
M-DPYS-py4	GGGATTTGAGTGTGAAAGGATTAT *	ACAACCCCCCAACTCTAACTC	159	60	TTTTTTAGGGGGAGG	3	AGTYGTATGTGYGGTTAGGTTATAAATTYGTTAGGT
M-GATA4-py1	ATGAGGGGGAGGGGAATATTAA	ACCCCTACCAAATTTCCAATCTTA	199	60	TTTAGGGTTAATTAGTTTGT	2	YGGYGATGTGTATTYGGTTGTATATTYGGGGAT
M-HOXA2-py1	TTTGGGAGTGTGTGTGTGTGAAT *	AACCCTCCAATATCAAAACTTTAAAAAT	215	65	TGAGAGTGTAAGTTTTGTTGT	3	YGGTTTYGTYGGTGGTT
M-KLF14-py1	TGTTGTAGTTAGGGAAGGGGTATTGGT	CCAACCCACCAACAACCTCTAAT	141	55	TTTTTTAGGGGTATTTGAT	5	TYGYGAYGGGGGYGGGGTYGGTTTTTA
M-LOC721190-py2	ATGTTGAGGGGAGAGTTAGGATTA *	CCAACACCCCCAAATCAC	112	60	TGGGGGGTTTTTGTT	2	TYGAGTTYGGGGGTGATTTGGGGGTGTTGG
M-MYOD1-py2	AGGGTAGTTGTTTATTGTGGGTTTGTAA	CCCTTCAACCTCTCAAAAACCTCAT	133	65	GGAGTGTAAGAGTAAGATTATTAA	5	YGTYGATYGTYGTAAGGTYGTTA
M-NPTX2-py1	AGGGGATAGGGATTAGGATTAGGTA	TCCATCCCCATAACTCCAATTAAAT	192	65	ATAGGGATTAGGATTAGGTAT	5	YGGAYGTTGAYGGAAAGGYGGGGATATTYGGAG
M-PGR-py2	TTTTGGGGTAGATTTTGGAGTGTA	CCCAACCCTCACCTCAAATAATT	292	60	GGAGTGTATTTTGTATAAAG	4	YGGAGGGYGYGTYGTTTTA
M-RIPK4-py1	GGGAAAAAAAAGTAAAGAAAAGTAGG *	CCACCTTCTCCCAACCTATA	327	60	AAAAGTAGGGTAGTTGATGA	3	GTAYGTYGAGGTTTTTTTTAYGTTTTGAA
M-SALL3-py1	AAGTGGGTTTAGGTTGTTTATTT	CCCAAACCAAAAAAAATCCA	132	60	AGAGTTTTGGAGTAGAGATT	6	TYGGGGGGYGGYGYGGAGGYGGAGGTATYGTGG
M-SORCS1-py7	GGAGAGGTGAGGATTAGGAGAA *	CCCAATACCAAACTCCTACTACCC	101	60	GGATTAGGAGAAGGTAGAA	4	YGGGGAGAGGGYGYGAGTYGGAGTTTT
M-SOX11-py2	GGAGAAAGGGAAAAGGAGAGT	CCCCCTTTATACCCAATCC	317	60	GGAAGGTTGTTGTTTTTAA	6	GYGTTATTTTTYGTYGGTTAGGGTYGTYGYGGGGTTA
M-SOX1-py2	GAGAGGGTATTGGAGAATTTTAGTGTATA	CCCCAACCAAATTTAAACAAAC	278	60	GTATTGGAGAATTTTAGTGTATAT	4	YGYGGTTAAGAGTYGATGYGGGGTA
M-SOX1-py3	TAGGGGAGGTTGGTGGGTTTA*	CACCCCTCCCCATTCTTC	144	65	GAGGTTGGTGGGTTTAT	3	YGGTATYGYGAGGAG
M-SOX1-py4	AGAGGAGAGAAGAATGGGGAGG *	CCCAACCCTCAACCTCTTTAACA	140	65	TAAAAAGAGGTAGAAATATA	6	YGTATTYGGYGTYGGTTYGGTTYGTA
M-TRHDE-py2	ATGGTAGAGGTGGGAGAGATTAGA *	ACCCCCCCTTAAAATACCCAA	118	60	TTAGTAGGAGGAAGTTTTAG	3	GGTYGYGAAGTTAYGG
M-TRHDE-py5	GAGGGGATTTTTTGTAGTTTGAG	CCCCAAAAAAATAAAATTCTCCAT	188	60	GGGATTTAGTAGGTTTAGTA	3	GTYGTYGGAGGAGGAGYGGGAGT
M-TRIM58-py3	GGTTGTGTGTAGTAAAGGGGGTTA *	CCCACCCCTACCAAATAAAA	212	60	AGGAGATTTATATTGAGTGA	7	GTYGYGTGGGYGGYGGGGAYGGYGYGGGGAG
M-VWCE-py2	GGGGTAGGTTTAGAATTGTTTAG *	ACCCCAAAAAAAAACAAACTTCA	173	65	TTATAGATAGGGGTTATGTT	5	YGGGGYGYGTAGTTTYGGGYGTTA
M-DOK2-py1	TTTGGTTTTTAGTAAGAAGGTATTGG *	CCCCTTCCAAACCTCACTATAT	196	60	AGTAAGAAGGTATTGGTGTT	5	TYGGGGGTTGTTGGTTTTTTYGTYGGTTTYGGTTATTYGTA
M-EIF4EBP3-py3	AGAGAGGGAGGGGTAGTAAAGTAA	CCCAATAACCCACCCTAAACATA	137	60	AAGTAATAGTTTGGTATTGA	2	GGTYGAGGGGYGTAGTTTTGAGTTTGA
M-GOLT1A-py1	GTGTGGGGAAAGATGTTTGAAT	TACTCAAAACCCACAAATAACCAA	142	60	GAGTTTATTTTTGTGGTAAT	1	TYGGGATGGGAAAGGGGTTAGTGTGA
M-LOC695235-py1	AGTGGAGGGTTTATGTGATGTGAG *	TAACATACACACCACCCCTTACCA	120	60	AAAGTTGAGAGTGTTTT	1	YGGGATAGTATGTAGTTGGTAAGGGGT
M-LOC701241-py3	GGTTTAGGAGGTGGGTAGAGTTT	CCCATACCAACAACCATTACAA	124	60	TGGGTAGAGTTTTAGGGTT	1	YGGGGAAAGGTTTTATGGGGTGGAATG
M-LOC703422-py1	AGAAGGAAGAGGGTAGGGAAGGA	ACTCCCCTCCTCCTCTTACCTAA	144	60	GGTAGGGAAGGAGGTT	3	YGGGTTTAYGYGGTTTGGGTATAG
M-LOC703422-py2	TGTTTGAGGAGTTTAGGAAAGGT	AAAAACCCAACCAATCTCCTACTT	180	60	TAGGAAGGTTTTTGAGTTA	3	GGGYGTTGTGTTTAAGTYGYGTGGATT
M-MSLN-py2	GTGGGTATTAGGTGGGGTTGT	CCCCATCACTCCATAACTAAATCT	183	60	GGAGAGGAGGGGATAGT	2	TYGGGGGTTTTGGGGGTYGGGGTTTTAG
M-MSLN-py3	AGGGAGAAGGTAGGGATAGGTAAA *	CCACAACCTCCTATTCCTACTCTT	129	60	GTAAAGTTTTGTTGAGATTT	2	YGGTTTTTAAGGTTTTYGGGTTGTTTT
M-NCS1-py2	AATGGGGGGTTTTGTTTTAAGT	ACCCCCTCCCCAAAAATAAA	125	60	ATAGAGGAGGGAGTGTGT	4	YGTYGGTYGTATTTTTTTGTTTYGGAGTTG
M-RILP-py1	GTTTAAAGGGTTGGGTTGGATA	TCCCCTACCTTAACCACATTTACT	154	60	GGTTGGGTTGGATATTAT	2	AGTYGTTTAGYGTAAGTTAGAGTTTTTAGAT
M-SORBS2-py1	TGGGGGTAGGTAGGTGGAAA	TTCTCATCCACAAACCCCTAAAA	118	65	GGTGGGGAGAGAGG	4	YGAGTTGGGGGAGYGGGATAGGGYGTYGGGGGGTTTTTA
M-FNBP1-py2	AGGGGATTAGTGATGATATTTTTATTAAAGG *	TACCAAACCATCTCTCTCTCTACAACA	277	60	TTATTAAAGGTATTGTGTGAAG	5	YGYGGAAGGGTYGTTAGGAYGGGTTYGGGTGGA
M-VSIG2-py1	GGGGATAGAGTGGGTTTTAAGGA	CAACTCCCCAAACCCCTTTC	140	60	GGTTTATGTTTTTTAGTTTT	4	YGTYGTGGTTTYGTTTGGYGTTTTTTTATTATTTAG
M-VSIG2-py2	GGGGATAGAGTGGGTTTTAAGG	CTCCCCAAACCCTTTCTCTAC	137	60	GAGTGGGTTTTAAGGAGT	1	TYGGGAAGTTAGGTTTATGTTTTTTAGT
Human							
H-AC009501-py6	GGAAGGTAGTGAGTTTTGGAATGT *	CCCCCAAAATAATAACAACATCTA	99	60	TGAGTTTTGGAATGTGTTAG	4	YGYGGTTTTYGGGGTAYGTTT
H-ALOX12-py5	GGATTTGGGAGAGTTTAAAATTTG	ATAACCCCCCCCAACAACTT	153	55	AAAATTTGAGAGGAGGG	4	YGGGGTYGTAGATYGGTTTTTTAAAGGTTGGAAGTGGTTTYGAG
H-HOXA2-py3	TGGTTAAGGGGATTTGGATATATG *	TACCCCCCCAACCCATTATTAAC	198	60	TTGATGGAGTAGGAAGGT	5	YGGGAAGTTTYGAGAGTYGTTTTTYGTTTTTYGTTTTT
H-KLF14-py2	GGGAGAATTGTTTGGGGTAGAG *	TCCCCAACTACACCAAAACCT	201	65	GAGGGGGTAGGAGAAG	5	YGTTTTTYGTTYGTGTGYGTTTTGTAGTGGYGGGTTA
H-NPTX2-py3	TGATGGGAAGGAGGGGATATT *	CCCCCCCTAAACTCAAATCTT	130	60	GAGGTTATTAGTAGAGTTTT	6	YGYGYGYGTGTTTATYGATTGTTTTTTGGGYGGTGA
H-RIPK4-py1	GGGTTATTAGGGGAGTGTTAGGAA *	CTCTAATCCCCAAATCCTACCTC	279	60	ATAGGTAGAGTGGATATTGT	6	YGGAGTTTYGYGGTTTYGTYGGTAATTGGTTTTTTTTTYGAAG
H-SORCS1-py4	GGAGAGGATTAGGAGAAGGTAGAA	CCTCCCCAATCCCAAACT	100	60	AGGTAGAAAGGGGAGAG	4	GGYGYGAGTYGGAGTTTTYGGGGAGTGTTA
H-SOX11-py1	AGGGTTTTTAGGGGATTGGGTAT *	TCCCCCCATCTTCCTTTATTAACT	295	60	GGGATTAGGTTTTAGGTTAG	8	YGTYGGYGTTYGAATTATTTYGGTTYGTTTYGGGTYGTT

H-MSLN-py1-F	GGGGTGGAGAGTTTGGTAGTTT *	TCCACCCACACATTCCTAAAA	109	60	GGTAGTTAAAGGGAATAAAT	2	GAYGGTTTTGTTYGGGGTAGTAGGGAGTGG
H-RILP-py1-F	TGGAGTTATGGGGTTAATTTGGTA *	TCCAACCCAACCCTTTAAACTTT	159	60	GGGTTAATTTGGTAGTGGT	2	TYGTTTTAGGYGAGAGGTGATAGGTAGGTAG

We used UNIV-reverse primer and Biotin-UNIV primer for 2nd PCR. Annealing temperature is same as 1st PCR.
*; forward and UNIV-reverse primer for 1st PCR, forward and Biotin-UNIV primer for 2nd PCR.
YG; CpG site for pyrosequencing analysis.

Supplementary Table 21 | Primers used for telomere length assays in mice, monkeys and humans.

Primer name	Primer sequence (5'-3')
Telomere-1-F	CGGTTTGTTTGGGTTTGGGTTTGGGTTTGGGTTTGGGTT
Telomere-1-R	GGCTTGCCTTACCCTTACCCTTACCCTTACCCTTACCCT
Telomere-2-F	GGTTTTTGAGGGTGAGGGTGAGGGTGAGGGTGAGGGT
Telomere-2-R	TCCCGACTATCCCTATCCCTATCCCTATCCCTATCCCTA
Mouse-36B4-F	ACTGGTCTAGGACCCGAGAAG
Mouse-36B4-R	TCAATGGTGCCTCTGGAGATT
Monkey-36B4-F	ATGTCAAGCACTTCAGGATTGTAG
Monkey-36B4-R	AACATCTCCCCCTTCTCCTTT
Human-HBG1-F	GCTTCTGACACAACTGTGTTCACTAGC
Human-HBG1-R	CACCAACTTCATCCACGTTCACC
